# Roles of DEMETER in regulating DNA methylation in vegetative tissues and pathogen resistance

**DOI:** 10.1111/jipb.13037

**Published:** 2021-03-16

**Authors:** Wenjie Zeng, Huan Huang, Xueqiang Lin, Chen Zhu, Ken‐ichi Kosami, Chaofeng Huang, Huiming Zhang, Cheng‐Guo Duan, Jian‐Kang Zhu, Daisuke Miki

**Affiliations:** ^1^ Shanghai Center for Plant Stress Biology and Center of Excellence for Molecular Plant Sciences the Chinese Academy of Sciences Shanghai 210602 China; ^2^ University of the Chinese Academy of Sciences Shanghai 201602 China; ^3^ Fruit Tree Research Center, Ehime Research Institute of Agriculture, Forestry and Fisheries Ehime 7910112 Japan; ^4^ Department of Horticulture and Landscape Architecture Purdue University West Lafayette Indiana 47907 USA

**Keywords:** active DNA demethylation, *Arabidopsis*, disease resistance, DME, DNA methylation

## Abstract

DNA methylation is an epigenetic mark important for genome stability and gene expression. In *Arabidopsis thaliana*, the 5‐methylcytosine DNA glycosylase/demethylase DEMETER (DME) controls active DNA demethylation during the reproductive stage; however, the lethality of loss‐of‐function *dme* mutations has made it difficult to assess DME function in vegetative tissues. Here, we edited *DME* using clustered regularly interspaced short palindromic repeats (CRISPR) /CRISPR‐associated protein 9 and created three weak *dme* mutants that produced a few viable seeds. We also performed central cell‐specific complementation in a strong *dme* mutant and combined this line with mutations in the other three *Arabidopsis* demethylase genes to generate the *dme ros1 dml2 dml3* (*drdd*) quadruple mutant. A DNA methylome analysis showed that DME is required for DNA demethylation at hundreds of genomic regions in vegetative tissues. A transcriptome analysis of the *drdd* mutant revealed that DME and the other three demethylases are important for plant responses to biotic and abiotic stresses in vegetative tissues. Despite the limited role of DME in regulating DNA methylation in vegetative tissues, the *dme* mutants showed increased susceptibility to bacterial and fungal pathogens. Our study highlights the important functions of DME in vegetative tissues and provides valuable genetic tools for future investigations of DNA demethylation in plants.

## Graphical abstract

The *Arabidopsis* demethylase DEMETER acts on some target loci in the vegetative stage and plays a vital role in positive regulation of the response to pathogen infection, acting synergistically with three other demethylases.

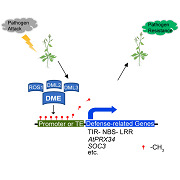

## INTRODUCTION

DNA methylation at cytosine residues is an important and conserved epigenetic modification in many eukaryotes, including plants, and is associated with the suppression of transposable elements (TEs) and the regulation of gene expression (Zhu, 2009; Zhang and Zhu, 2012; Zhang et al., 2018; Liu and Lang, 2020). In plants, DNA methylation occurs in three sequence contexts: CG, CHG, and CHH (H represents A, T, or C). In *Arabidopsis thaliana*, CG and CHG methylation is maintained by DNA METHYLTRANSFERASE 1 (MET1) and CHROMOMETHYLASE 3 (CMT3), respectively. Depending on the chromatin context, CHH methylation is maintained by CMT2 or by DOMAIN REARRANGED METHYLTRANSFERASES (DRM1 and DRM2) through the RNA‐directed DNA methylation pathway, which is also responsible for *de novo* DNA methylation.

DNA methylation levels are determined through the balanced regulation of establishment, maintenance, and removal activities. DNA methylation can be passively lost due to defective maintenance or can be actively removed through enzymatic reactions. In plants, active DNA demethylation is initiated by the 5‐methylcytosine DNA glycosylase enzymes. There are four 5‐methylcytosine DNA glycosylase genes in *Arabidopsis*: *REPRESSOR OF SILENCING 1* (*ROS1*), *DEMETER* (*DME*), *DEMETER‐Like 2* (*DML2*), and *DML3* (Penterman et al., 2007). The roles of *ROS1*, *DML2*, and *DML3* in vegetative tissues have been characterized; in contrast, not much is known about *DME* function in vegetative tissues due to the seed‐abortion phenotype of the *dme* loss‐of‐function mutants (Choi et al., 2002). *DME* is expressed in the central cell of the female gametophyte and is required for gene imprinting in the endosperm (Choi et al., 2002). A recent study indicated that *DME* is expressed not only in the central cell but also in vegetative tissues (Park et al., 2017; Schumann et al., 2019). During the preparation of this manuscript, Schumann et al. (2019) reported using a β‐glucorinidase (GUS) reporter gene and quantitative real‐time polymerase chain reaction (qRT‐PCR) to show that *DME* is expressed in vegetative tissues, and found that *Arabidopsis* plants with a RNA interference (RNAi)‐mediated knockdown of *DME* expression showed increased susceptibility to the fungal pathogen *Fusarium oxysporum*. However, the lack of stable and viable genetic *dme* mutants in that study hindered the analysis of the genetic function of *DME* in regulating the DNA methylome, gene expression, and responses to the environment.

In this study, we generated stable genetic materials for studying *DME* function in vegetative tissues. These genetic materials included three weak *dme* mutants and central cell‐specific *DME* complementation mutant lines from which quadruple DNA demethylase mutants were subsequently obtained. The analyses of the transcriptomes and DNA methylomes in the *dme* mutants indicated important roles for DME in regulating genomic DNA methylation in vegetative tissues, particularly in genes related to biotic stress responses. We show that DME plays a key role in resistance against the fungal pathogen *Verticillium dahliae*, a finding consistent with Schumann et al. (2019), and against the bacterial pathogen *Pseudomonas syringae pv. tomato* (*Pst*).

## RESULTS

### *DME* expression in vegetative tissues

*DME* was originally thought to be specifically expressed in the central cell to control gene imprinting (Choi et al., 2002); however, public *Arabidopsis* transcriptome databases (AtGenExpress Visualization Tool, eFP Browser, and TraVA) indicate that *DME* is also constitutively expressed in *Arabidopsis* vegetative tissues (Figure S1A) (Schmid et al., 2005; Mathieu et al., 2007; Klepikova et al., 2016). Using a GUS reporter gene and qRT‐PCR, two groups have found that the *DME* promoter has transcriptional activity in somatic cells (Park et al., 2017; Schumann et al., 2019). We also performed qRT‐PCR to assess the expression of all DNA demethylases including *DME* in leaf tissues of wild‐type *Arabidopsis* (Col‐0 accession), as well as some DNA demethylase and methyltransferase mutants (Figure S1B). Our results provide further evidence that *DME* is expressed in the leaf tissues (Figure S1B). As previously reported, *ROS1* expression was downregulated in the DNA methyltransferase mutants (Huettel et al., 2007; Mathieu et al., 2007; Martínez‐Macías María et al., 2012; Lei et al., 2015). Although *DME* expression was previously reported to also be suppressed in *met1* and *drm2* mutants (Mathieu et al., 2007), we found that the *DME* expression level was not altered in these mutant backgrounds under our experimental conditions (Figure S1B). These results support that *DME* is expressed in vegetative tissues, and suggest that unlike *ROS1*, *DME* expression is not responsive to genomic DNA methylation status.

### Generation of *dme* mutants

**Figure 1:**
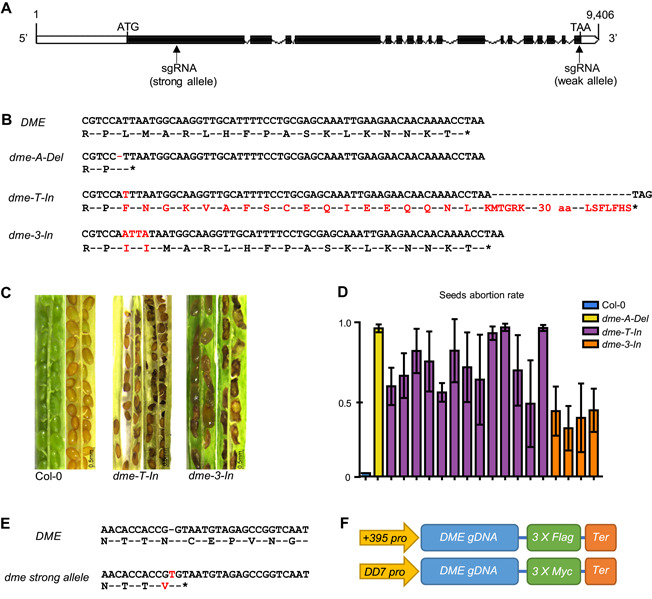
Generation of *dme* mutants (**A**) Schematic diagram of the *DME* gene structure. The boxed regions are exons, and the lines are introns. The arrows indicate the positions of the single guide RNA (sgRNA) target sites for the *dme* weak‐ and strong‐allele mutants. (**B**) Partial DNA and amino acid sequences of three *dme* weak‐allele mutants: *dme‐A‐Del, dme‐T‐In*, and *dme‐3‐In*. Nucleotides and amino acid residues not present in the wild type are shown in red. (**C**) Seed‐abortion phenotype. Opened siliques from Col‐0 plants and homozygous *dme‐T‐In* and *dme‐3‐In* mutant plants. Scale bar, 0.5 mm. (**D)** Proportion of aborted seeds. One *dme‐A‐Del*, 13 *dme‐T‐In*, and four *dme‐3‐In* mutant plants were analyzed (five siliques per plant). (**E**) Partial DNA and amino acid sequences of the *dme* strong‐allele mutant. (**F**) Constructs for central cell‐specific complementation. The *DD7* promoter controls gene expression in a central cell‐specific manner, and the +395 to +1967 sequence of the *DME* 5′ untranslated region (*+395 pro*) contributes to the strong *DME* expression in the central cell only.

A complete loss of DME function causes maternal mortality, which has hindered investigations of DME functions (Choi et al., 2002; Tsuzuki et al., 2014; Yang et al., 2016). To analyze the function of this 5‐methylcytosine DNA glycosylase in vegetative tissues, we first generated weak *dme* mutants using clustered regularly interspaced short palindromic repeats (CRISPR)/CRISPR‐associated protein 9 (Cas9). In the three weak *dme* mutants generated, the mutations were located in the last exon to mitigate nonsense‐mediated messenger RNA decay (Figure 1A). The first mutant allele, named *dme*‐*A*‐*Del*, contains a single adenine deletion that results in a premature stop codon (Figure 1B). The second allele, *dme*‐*T*‐*In*, contains a thymine insertion that generates extra peptide sequences (Figure 1B). The third mutant allele, *dme*‐*3*‐*In*, contains a single‐nucleotide deletion and a four‐base insertion that cause a one‐amino‐acid substitution and a one‐amino‐acid insertion (Figure 1B). Although these three weak‐allele mutants showed a strong seed‐abortion phenotype, a few seeds survived and could germinate (Figure 1C, D). The *dme*‐*A*‐*Del* mutant showed the strongest seed‐abortion phenotype, producing almost no seeds (Figure 1D). The *dme*‐*3*‐*In* mutant showed the weakest seed‐abortion phenotype (Figure 1D). In these *dme* weak‐allele mutants, there were no morphological phenotypes other than seed abortion. An analysis of the gene expression of the other DNA demethylases in these mutants revealed that the expression of *DML2* and *DML3* was not changed, whereas *ROS1* expression was slightly elevated in the strongest *dme‐A Del* mutant (Figure S2).

According to a previous report, the DNA demethylases ROS1, DML2, and DML3 are functionally redundant in *Arabidopsis* (Penterman et al., 2007). To analyze DME function, we attempted to generate a *dme ros1 dml2 dml3* (*drdd*) quadruple mutation in the *ros1 dml2 dml3* (*rdd‐2*) triple mutant background but failed because of embryo lethality. This result may indicate that, in addition to DME, the other DNA demethylases are required for normal embryo development. We therefore altered our strategy and generated central cell‐specific complementation lines in the *drdd* mutant background, which harbors a strong *dme* mutant allele in addition to the *rdd‐2* triple mutations in the Col‐0 background (Yamamuro et al., 2014). This *rdd‐2* mutant is different from the previously reported *rdd* triple mutant (Penterman et al., 2007), which harbored six‐times‐backcrossed *ros1‐3* and *dml2‐1* alleles in the Ws‐0 background.

First, we generated a strong *dme* mutant using CRISPR/Cas9 to target the first exon of *DME* (Figure 1A). The generated mutant contained one T insertion, which resulted in a premature stop codon (Figure 1E). This mutant had a strong seed‐abortion phenotype, and no homozygous mutant was obtained, consistent with it being a strong *dme* allele. The seed‐abortion phenotype in the *dme* mutants is caused by a loss‐of‐function in the gametogenesis stage, resulting in abnormal embryo development (Choi et al., 2002). To generate a viable *dme* mutant for studying *DME* in vegetative tissues, we therefore specifically expressed *DME* in the central cell using the *DD7* and *DME* + *395* promoters. The *DD7* promoter controls gene expression in a central cell‐specific manner (Steffen et al., 2007), and the +395 to +1967 sequence of the *DME* 5′ untranslated region (UTR) contributes to the strong expression of *DME* in the central cell (Park et al., 2017). These two promoters were independently fused with the full‐length genomic sequence of *DME* (ATG to TAA) (Figure 1F), and the constructs were transformed into the heterozygous strong *dme* mutant. We then screened for homozygous strong mutant lines with the *DME* functional complementation in a central cell‐specific manner. Transgenic *DME* expression was analyzed in T1 generation leaves using qRT‐PCR, and transgenic lines with an undetectable level of *DME* transcripts in the vegetative tissues were then crossed with *rdd‐2* to obtain *drdd* ^*DD7 pro*^ and *drdd* ^*+395 pro*^, which are quadruple mutants with central cell‐specific *DME* complementation. Morphological defects and seed‐abortion phenotypes were not observed in the *dme* ^*DD7 pro*^ single mutant or in the *drdd* ^*DD7 pro*^ or *drdd* ^*+395 pro*^ quadruple mutants.

### Pruning of DNA methylation by DME in vegetative tissues

**Figure 2:**
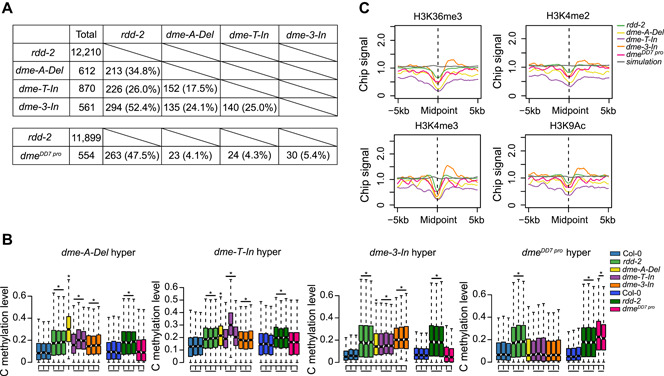
Characterization of *dme* single mutants (**A**) Overlap of hyper‐differentially methylated regions (hyper‐DMRs) among *rdd*‐*2*, *dme‐A‐Del*, *dme‐T‐In*, *dme‐3‐In*, and *dme* ^*DD7 pro*^ mutants. Two independent methylome analyses, one for *dme* weak‐allele mutants and the other for *dme* ^*DD7 pro*^ and *rdd‐2* samples, were performed. (**B**) Box plots of the *dme‐A‐Del*, *dme‐T‐In*, *dme‐3‐In*, and *dme* ^*DD7 pro*^ mutant‐specific hyper‐DMRs. DNA methylation levels of *dme‐A‐Del*, *dme‐T‐In*, *dme‐3‐In*, and *dme* ^*DD7 pro*^ mutant‐specific hyper‐DMRs in mC are shown for Col‐0, *rdd‐2,* and four *dme* weak‐allele mutants, with replicates displayed in the same color. The levels in the mutants were analyzed relative to Col‐0 (*P < 10^–5^ compared with Col‐0, one‐tailed Wilcoxon tests). (**C**) Association of different histone modifications at regions surrounding hyper‐DMRs in four *dme* single mutants. The controls are associations of histone modifications at simulated regions with random genomic sequences of the same length distribution as the hyper‐DMRs of *rdd‐2*.

To determine the function of DME in vegetative tissues, we performed whole‐genome bisulfite sequencing of 10 d old seedlings of the three *dme* weak‐allele and *dme* ^*DD7 pro*^ single mutants. Most samples were represented by three replicates, and the average depth of coverage for each replicate was >10. We also used the *rdd‐2* triple mutant as a control. Using a method based on Fisher's exact test, we identified 612, 870, 561, and 12 210 hypermethylated differentially methylated regions (hyper‐DMRs) in the *dme*‐*A*‐*Del*, *dme*‐*T*‐*In*, *dme*‐*3*‐*In*, and *rdd*‐*2* mutants, respectively. Further, we performed another batch of independent whole‐genome bisulfite sequencing and identified 554 and 11 899 hyper‐DMRs in the *dme* ^*DD7 pro*^ single and *rdd*‐*2* mutants, respectively (Figure 2A). As a comparison, 6 902 hyper‐DMRs and 495 hypo‐DMRs were previously reported in the *ros1‐4* mutant (Tang et al., 2016). These results indicate that DME plays a role in DNA demethylation in vegetative tissues, although this role is minor compared with that of ROS1. Approximately 26.0%–52.4% of the hyper‐DMRs in *dme* weak‐allele mutants overlapped with those in *rdd‐2* (Figure 2A). Further examination of the methylation levels in CG, CHG, and CHH contexts revealed that, at these hyper‐DMRs in the *dme* single mutants, the hypermethylation mainly occurs in CG and CHG contexts, with less change in CHH methylation (Figures 2B, S3A). A heatmap of DNA methylation also indicated that some hyper‐DMRs in the *dme* single mutants overlapped with those in *rdd‐2*, while some may be specific to each mutant (Figure S3B).

To determine which histone marks are associated with DME targets, we used the publicly available data from the Gene Expression Omnibus (Accession No. GSE28398) (Luo et al., 2013), and analyzed histone features as previously described (Tang et al., 2016). The DME targets showed a statistically significant negative association with most of the active histone marks, such as H3K36 trimethylation (H3K36me3), H3K4me2/3, and H3K9 acetylation (H3K9ac), compared with the control regions (randomly selected genomic regions with the same length distribution as the DMRs). The negative correlation was greater in *dme* weak‐allele mutants, especially in *dme*‐*T*‐*In* and *dme*‐*A*‐*Del*, than in *rdd‐2* (Figure 2C).

**Figure 3:**
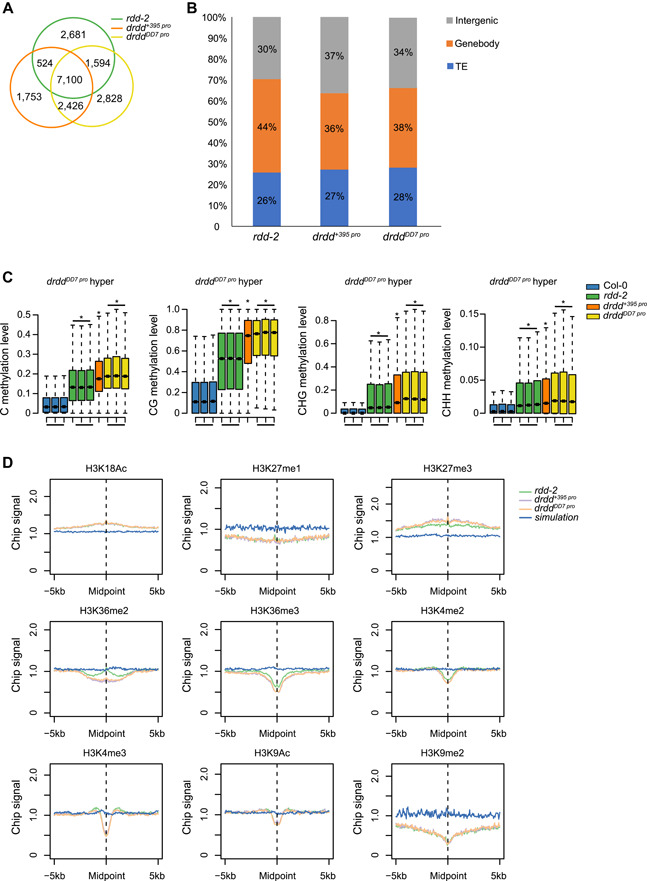
Characterization of central cell‐specific complementation *drdd* quadruple mutants (**A**) Overlap of hyper‐differentially methylated regions (hyper‐DMRs) among *rdd*‐*2, drdd* ^*+395 pro*^, and *drdd* ^*DD7 pro*^ mutants. (**B**) Distribution of hyper‐DMRs at transposable elements (TEs), and intergenic and gene body regions in *rdd*‐*2, drdd* ^*+395 pro*^, and *drdd* ^*DD7 pro*^ mutants. (**C**) Box plots of hyper‐DMRs specific to the *drdd* ^*DD7 pro*^ quadruple mutant. DNA methylation levels of the *drdd* ^*DD7 pro*^‐specific hyper‐DMRs in mC, mCG, mCHG, and mCHH contexts are shown for Col‐0, *rdd‐2*, *drdd* ^*+395 pro*^, and *drdd* ^*DD7 pro*^ mutants, with replicates displayed in the same color. The levels in the mutants were analyzed relative to Col‐0 (*P < 10^−8^ compared with Col‐0, one‐tailed Wilcoxon tests). (**D**) Association of different histone modifications at regions surrounding hyper‐DMRs in *drdd* quadruple mutants. The controls are associations of histone modifications at simulated regions with random genomic sequences of the same length distribution as the hyper‐DMRs of *rdd‐2*.

Using the same analysis pipeline, we identified 11 803 hyper‐DMRs in *drdd* ^*+395 pro*^ and 13 948 in *drdd* ^*DD7 pro*^. More than 60% of these hyper‐DMRs (64.6% for *drdd* ^*+395 pro*^ and 62.3% for *drdd* ^*DD7 pro*^) overlapped with the hyper‐DMRs in *rdd‐2* (Figure 3A), while 35.4%–37.7% of the hyper‐DMRs appeared to be specific to the *drdd* quadruple mutants (Figure 3A). The distribution of hyper‐DMRs in the *drdd* quadruple mutants was similar to that in *rdd‐2*, although the distribution ratio was slightly decreased in the gene body in both *drdd* quadruple mutants (Figure 3B). At the hyper‐DMRs of *drdd* ^*DD7 pro*^ mutant, an increase in DNA methylation in all cytosine contexts was also observed in *rdd‐2*, and a higher increase was observed in the other *drdd* quadruple mutant (Figures 3C, S4A). A heatmap analysis showed that DNA methylation levels at some genetic loci were increased in the *drdd* quadruple mutants but not in *rdd‐2* (Figure S4B). Because ROS1‐targeted TEs tend to be located near genes (Tang et al., 2016), we also analyzed the distance between the TEs and genes, and found that both *dme*‐ and *drdd*‐affected TEs were closer to genes compared with the unaffected TEs (Figure S4C). Together, these results indicate that the four DNA demethylases have functional redundancy, and that DME has a role in regulating DNA methylation in *Arabidopsis* vegetative tissues.

We next determined which histone modifications may be associated with hyper‐DMRs in *drdd* quadruple mutants. The *drdd* and *rdd‐2* hyper‐DMRs were positively associated with the repressive histone mark H3K27me3 and the active histone mark H3K18ac, but were negatively associated with the active histone marks H3K36me3, H3K36me2, H3K4me2/3, and H3K9ac, in contrast to the control regions (Figure 3D). In addition, the *drdd* and *rdd‐2* hyper‐DMRs were negatively associated with the repressive histone modifications H3K9me2 and H3K27me1, unlike the control regions (Figure 3D). In these associations with histone modifications, the hyper‐DMR peaks in *drdd* quadruple mutants largely behaved like those in *rdd‐2* (Figure 3D), which is consistent with the predominant role of ROS1 in regulating DNA methylation in vegetative tissues. By contrast, the positive association between hyper‐DMRs and H3K27me3 and the negative associations between hyper‐DMRs and H3K36me3, H3K4me2, and H3K36me2 are enhanced in the *drdd* quadruple mutants compared with the *rdd‐2* mutant (Figure 3D). These results indicate that DME has distinct genomic targets in vegetative tissues.

### DME regulates gene expression in vegetative tissues

**Figure 4:**
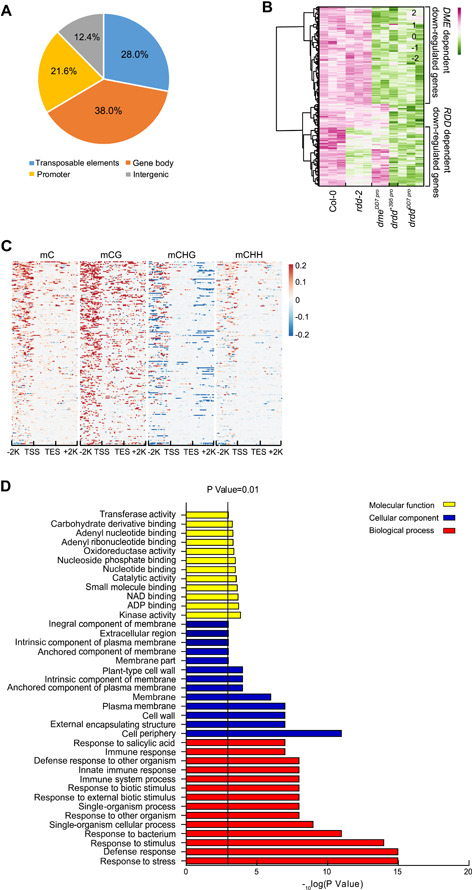
Methylation and transcriptome analyses in *drdd* quadruple mutants (**A**) The distribution of hyper‐differentially methylated regions (hyper‐DMRs) in the *drdd* ^*DD7 pro*^ quadruple mutant. (**B**) Heatmap analysis of transcript levels of 205 hyper‐DMR‐associated downregulated differentially expressed genes (DEGs) in the *drdd* ^*DD7 pro*^ quadruple mutant. Transcript levels in *rdd‐2*, *dme* ^*DD7 pro*^, *drdd* ^*+395 pro*^, and *drdd* ^*DD7 pro*^ are shown. (**C**) DNA methylation changes surrounding the 205 downregulated DEGs in the *drdd* ^*DD7 pro*^ quadruple mutant. (**D**) Gene Ontology (GO) analysis of the downregulated DEGs with hyper‐DMRs in the promoter regions in the *drdd* ^*DD7 pro*^ quadruple mutant.

The distribution of hyper‐DMRs in *drdd* ^*DD7 pro*^ is shown in Figure 4A, with approximately 21.6% mapped to promoter regions. Both DME‐ and DRDD‐targeted TEs are located near genes (Figure S4C). In the central cell, DME regulates the expression of genes, such as *MEA* and *FWA*, through the DNA demethylation of their regulatory regions (Choi et al., 2002; Gehring et al., 2006). Researchers have also found that the expression of many genes was altered in *ros1* and *rdd* mutant seedlings (Zhu et al., 2007; Lister et al., 2008; Stroud et al., 2013). Those results indicate that DME might also participate in the regulation of gene expression in vegetative tissues. We therefore performed a transcriptome analysis of the *dme* ^*DD7 pro*^, *drdd* ^*+395 pro*^, and *drdd* ^*DD7 pro*^ mutants, including *rdd‐2* as a control, to determine whether DME regulates gene expression in vegetative tissues. The expression of many genes was decreased in the quadruple mutants, while the single mutant also displayed a decreased expression of some genes. The *drdd* ^*+395 pro*^ and *drdd* ^*DD7 pro*^ mutants shared 208 downregulated differentially expressed genes (DEGs), which represented 38.4% of the downregulated DEGs in *drdd* ^*+395 pro*^ and 35.6% of the downregulated DEGs in *drdd* ^*DD7 pro*^ (Figure S5A). A total of 245 genes were downregulated in *rdd‐2*, a number lower than in the quadruple mutants. There were a large number of genes with decreased expression in the *drdd* quadruple mutants and in the *dme* ^*DD7 pro*^ single mutant, but their expression was not substantially altered in *rdd‐2* (Figure S5B). These results indicate that DME is required for the expression of a large number of genes in vegetative tissues.

To analyze the relationship between DNA methylation and gene expression, we first identified 6 943 genes with hyper‐DMRs in the promoter region in *drdd*, as revealed from the hyper‐methylation data. We then analyzed the expression levels of these genes, and found that while the expression of the majority of these genes with promoter hyper‐DMRs (6 654) was not changed, the expression levels of 84 of these genes were increased, while 205 had decreased expression levels. Of the 205 downregulated genes, 111 were mainly regulated by DME since their expression decreased in *dme* ^*DD7 pro*^ but not *rdd‐2*, and 42 were redundantly regulated by the demethylases because they displayed a very strong downregulation in the quadruple mutants only (Figure 4B). The DNA methylation levels of the promoter regions of the 205 downregulated DEGs in the *drdd* ^*DD7 pro*^ mutant were elevated relative to Col‐0, especially in the CG context (Figure 4C). There appeared to be a decrease in CHG methylation in these downregulated DEGs, especially at their promoters and downstream regions, even though the total cytosine methylation was increased (Figure 4C). These results show a strong correlation between DNA hypermethylation at the promoter regions and the downregulation of gene expression in the *drdd* mutants, indicating that active DNA demethylation is critical for the expression of these genes.

We performed a Gene Ontology (GO) analysis of the 205 downregulated DEGs associated with hyper‐DMRs in *drdd* ^*DD7 pro*^. These downregulated genes were highly enriched in the GO terms “response to stress”, “defense response”, “response to stimulus,” and “response to bacterium” (Figure 4D). Our analysis therefore suggests that demethylase‐dependent DNA demethylation may contribute to biotic and abiotic stress responses in vegetative tissues.

### Susceptibility of *dme* mutants to the bacterial pathogen *Pst* DC3000

**Figure 5:**
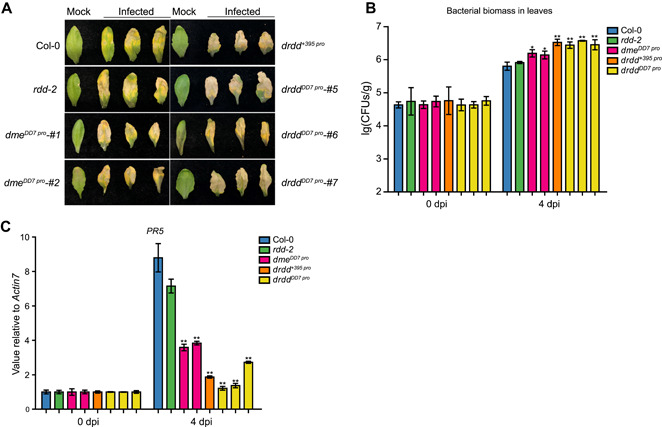
*Pst* DC3000 infection phenotype in *dme*^*DD7 pro*^ and *drdd* quadruple mutants (**A**) Symptoms caused by *Pst* DC3000 on leaves of *dme* single and *drdd* quadruple mutants. The leaves were inoculated with *Pst* DC3000 using a syringe. Col‐0 and *rdd‐2* were used as controls. Pictures were taken after 4 d post‐infection (dpi). (**B**) *Pst* DC3000 colony‐forming units (CFUs) in leaves of *dme* single and *drdd* quadruple mutants. The data were plotted as the mean ± *SEM*. Asterisks indicate significant differences when compared with Col‐0 (*P < 0.05, **P < 0.01, one‐tailed Student's *t*‐test). (**C**) Expression of *PR5* in *dme*, *rdd‐2*, and *drdd* mutants. The *PR5* expression was determined using a quantitative real‐time polymerase chain reaction (qRT‐PCR) with Col‐0 as a control, and the data shown are means ± *SEM* from three experiments (*P < 0.05, **P < 0.01, one‐tailed Student's *t*‐test).

The methylome and transcriptome analyses suggested that the DNA demethylases, including DME, might be involved in biotic stress responses (Figure 4D). We therefore determined the effects of the bacterial pathogen *Pst* DC3000 on the *dme* weak‐allele mutant and the central cell‐specific complementation *dme* and *drdd* mutants. In response to *Pst* DC3000 inoculation, all *dme* and *drdd* mutants showed a hypersensitive phenotype, including severe water‐soaked lesions and chlorosis, compared with that of *rdd‐2* and Col‐0 (Figures 5A, S6A). A hypersensitive phenotype was also observed in a plate assay using *Pst* DC3000 and detached leaves of the *dme* mutants (Figure S6B). Measurements of the bacterial titer in infected plants confirmed that the *drdd* quadruple mutants were more susceptible than the control Col‐0 or *rdd‐2,* and the bacterial count was also greater in the *dme* single mutant than in the *rdd‐2* mutant at 4 d post‐infection (dpi) (Figures 5B, S6C). The expression of a disease‐response marker gene, *PATHOGENESIS‐RELATED PROTEIN* 5 (*PR5*), was suppressed in the *dme* and *drdd* mutants (Figures 5C, S6D), although the methylation level of the *PR5* locus was not altered in the mutants. Further, a strong correlation was observed between the increased bacterial count and reduced *PR5* expression in the *dme* and *drdd* mutants (Figures 5B, C, S6C, D). Although ROS1 and RDD have been reported to be important for plant defense responses in *Arabidopsis* (Yu et al., 2013; Le et al., 2014; López Sánchez et al., 2016), *rdd‐2* did not show a great sensitivity to *Pst* DC3000 infection under our experimental conditions, even though *PR5* expression was lower in *rdd‐2* than in Col‐0 (Figures 5C, S6D). This result indicates that the four DNA demethylases redundantly regulate plant resistance to bacterial infection in vegetative tissues, and that DME is more important than the other three DNA demethylases for disease resistance.

The treatment of *Arabidopsis* with the bacterial flagellin‐derived peptide flg22 was shown to induce the downregulation of *ROS1* expression in the early stages of infection (Yu et al., 2013); therefore, we explored whether *DME* expression was altered by a flg22 treatment in Col‐0. Under our experimental conditions, the expression levels of *DME* and *ROS1* in Col‐0 were not greatly affected by the flg22 treatment (Figure S6E, F).

### Susceptibility of *dme* mutants to the fungal pathogen *V. dahliae*

**Figure 6:**
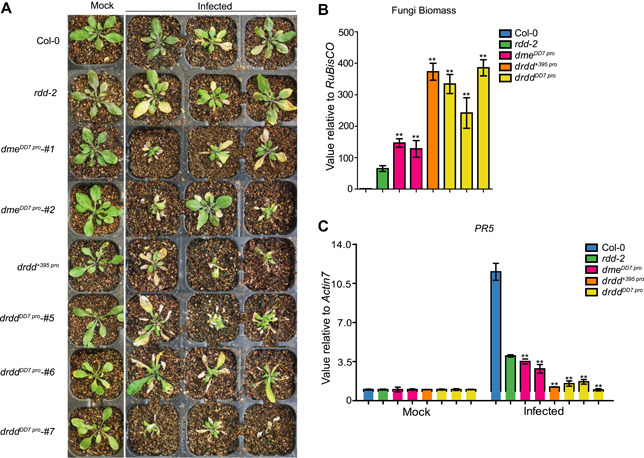
*Verticillium dahliae* infection phenotype in *dme*^*DD7 pro*^ and *drdd* quadruple mutants (**A)** Leaf symptoms caused by root dipping *rdd‐2*, *dme* single, and *drdd* quadruple mutants at 26 d post‐inoculation with *V. dahliae* strain V592, with Col‐0 as a control. (**B**) Transcriptional quantification of *V. dahliae* infection. The *V. dahliae* was quantified using real‐time polymerase chain reaction (qRT‐PCR) by comparing the *V. dahliae* internal transcribed spacer (ITS) expression levels (as a measure for fungal mass) relative to *Arabidopsis* RuBisCO levels in the leaves of Col‐0, *rdd‐2*, *dme*, and *drdd*, with Czapek–Dox liquid medium dipping as the mock, at 26 d post‐inoculation (**P < 0.01, one‐tailed Student's *t*‐test). (**C**) Expression of *PR5* was determined by qRT‐PCR in *rdd‐2*, *dme* single and *drdd* quadruple mutants with Col‐0 as a control, and data shown are means ± *SEM* from three experiments (**P < 0.01, one‐tailed Student's *t*‐test).

Researchers have previously found that the *rdd* mutant exhibits a hypersensitive response (HR) to the fungal pathogen *F. oxysporum* (Le et al., 2014; Schumann et al., 2017), and that *DME* contributes to the resistance against *F. oxysporum* (Schumann et al., 2019). As noted above, we found that *DME* is required for resistance to bacterial infection (Figures 5, S6); therefore, we next determined whether *DME* contributes to plant resistance to infection by the fungus *V. dahliae* strain V592. The roots of seedlings of the weak‐allele mutants and the central cell‐specific complementation single and quadruple mutants were inoculated with *V. dahliae* as previously described (Broekaert et al., 1990; Ellendorff et al., 2009). In response to inoculation, the *drdd* quadruple mutants were severely stunted and wilted; the *dme* single mutants had slightly less severe symptoms than the quadruple mutants but showed more severe symptoms compared with *rdd‐2* or Col‐0 (Figures 6A, S7A). The amount of *V. dahliae* present in the aboveground tissues (as indicated with a qPCR quantification of *V. dahliae* DNA) was consistent with the disease phenotypes; much more *V. dahliae* was present in the quadruple mutants than in the *dme* single mutants (Figures 6A, B, S7A, B). The aboveground *V. dahliae* contents were also higher in the *dme* single mutants than in the control Col‐0 or *rdd‐2* (Figures 6B, S7B). The expression of the disease‐response marker gene *PR5* was suppressed in the *dme* single and quadruple mutants (Figures 6C, S7C). The *V. dahliae* content was negatively correlated with *PR5* expression in the *dme* weak‐allele and quadruple mutants (Figures 6B, C, S7B, C). These results show that the impairment of the DNA demethylases reduces plant resistance to *V. dahliae*, and that DME plays a more prominent role than the other three demethylases in mediating *Arabidopsis* resistance to *V. dahliae*.

### DME is required for the expression of defense‐related genes

**Figure 7:**
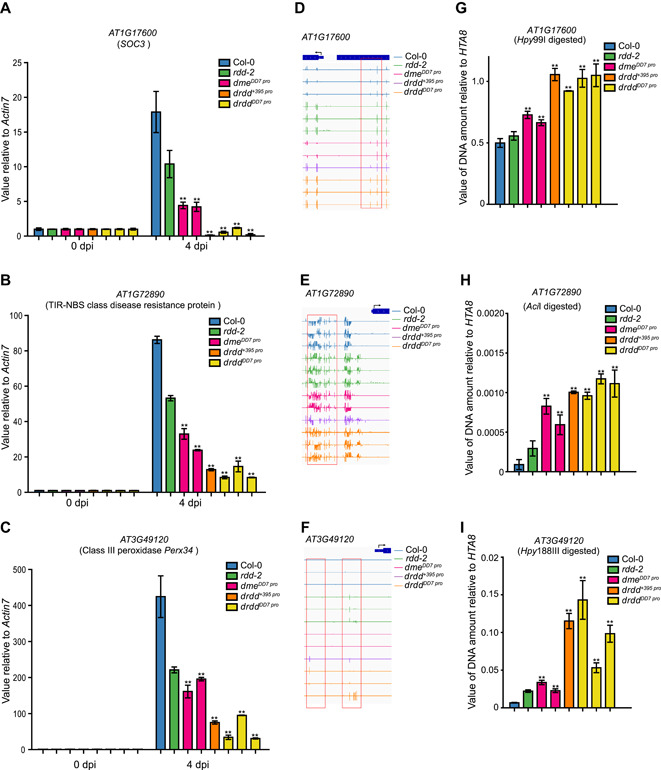
Expression analysis for defense‐related genes in *Pst* DC3000‐infected *dme*^*DD7 pro*^ and *drdd* quadruple mutants (**A**–**C**) The expression levels of three hyper‐differentially methylated region (hyper‐DMR)‐associated downregulated defense‐response genes (At1g17600 (*SOC3*; A), encoding a Toll‐interleukin‐1 receptor (TIR)‐NB‐leucine‐rich repeat (TNL) protein; At1g72890 (**B**), encoding a TIR‐NBS‐LRR class disease resistance protein; and At3g49120 (**C**), encoding the class III peroxidase *PERX34*) were analyzed in *rdd‐2*, the central cell‐specific complementation *dme* single mutant, and *drdd* quadruple mutants with Col‐0 as a control. The data shown are means ± *SEM* from three experiments (**P < 0.01, one‐tailed Student's *t*‐test). (**D**–**F**) Integrative Genomics Viewer (IGV) images showing the methylation status for three defense‐related genes in *dme* single and *drdd* quadruple mutants. DNA methylation levels of cytosines are indicated by the heights of the vertical bars on each track. Biological replicates are shown in the same color. (**G**–**I**) Quantitative Chop polymerase chain reaction for the promoters of three defense‐related genes in *dme* single and *drdd* quadruple mutants. DNA digested using the cytosine methylation‐sensitive restriction enzymes *Hpy*99I, *Aci*I, and *Hpy*188III was used as the template. The data shown are means ± *SEM* from three replicates (**P < 0.01, one‐tailed Student's *t*‐test).

Our transcriptome data for the *drdd* ^*DD7 pro*^ quadruple mutant (Figure 4D) and the results of our pathogen infection assays (Figures 5, 6) showed that *DME* and the other DNA demethylases regulate biotic stress responses in *Arabidopsis*. To further test the role of the DNA demethylases, we performed a qRT‐PCR with primers for the following three defense‐response genes, which corresponded to hyper‐DMRs in the *drdd* mutant plants (Figure 4B) and were listed as downregulated defense‐response genes in the GO analysis (Figure 4D): At1g17600 (*SOC3*), encoding a Toll‐interleukin‐1 receptor (TIR)‐NB‐leucine‐rich repeat (TNL) protein; At1g72890, encoding a TIR‐NBS‐LRR class disease resistance protein; and At3g49120, encoding the class III peroxidase *PERX34* (AtPRX34). SOC3 and the TIR‐NBS‐LRR protein are nucleotide‐binding leucine‐rich repeat (NLR) receptors that recognize pathogens either directly or indirectly (Monteiro and Nishimura, 2018), while AtPRX34 plays an important role in generating reactive oxygen species (ROS) during the defense response (Bindschedler et al., 2006; Daudi et al., 2012). All three genes were strongly suppressed in the central cell‐specific complementation single and quadruple mutants (Figure 7A–C) and in the *dme* weak‐allele mutants (Figure S8) when their leaves were infected with *Pst* DC3000. In plants without pathogen infection, the DNA methylation at these three loci was increased in not only the quadruple mutants but also the *dme* single mutant, although the increases were not large (Figure 7D–F). We performed a real‐time quantitative Chop‐PCR (qChop‐PCR) to analyze whether the DNA methylation levels at the promoter regions of the defense‐related genes may be increased in the mutants when the plants are infected by *Pst* DC3000. The results showed that DNA methylation levels at these loci were significantly increased in pathogen‐infected demethylase mutants, especially in the *dme* single and quadruple mutants, compared with the wild type (Figure 7G, H). These results clearly show that DME plays an important role in the regulation of the expression of defense‐related genes and in pathogen resistance (Figure S9).

## DISCUSSION

*DME* was originally thought to be specifically expressed in the central cell and the progenitor of the endosperm, where it controls the imprinting of genes such as *FWA* and *MEA* (Choi et al., 2002). However, public gene expression databases and recent publications now indicate that *DME* is ubiquitously expressed in various *Arabidopsis* organs and tissues (Schmid et al., 2005; Mathieu et al., 2007; Klepikova et al., 2016; Park et al., 2017; Schumann et al., 2019). Using RNAi‐mediated knockdown, Schumann et al. (2019) recently showed that *DME* expression in vegetative tissues contributes to the resistance against the fungal pathogen *F. oxysporum*. We also attempted to generate *DME* RNAi transgenic plants using a strong constitutive *35 S* promoter to drive the expression of trigger hairpin double‐stranded RNA (dsRNA), but we could not obtain any RNAi‐mediated knockdown plants. Perhaps the *35 S* promoter was active in the central cell and/or the vegetative cell of the pollen. Schumann et al. (2019) used a green tissue‐specific *Arabidopsis* RuBisCO small subunit (*SSU*) gene promoter to drive the expression of the trigger hairpin dsRNA. They obtained fully fertile but strongly downregulated *DME* RNAi lines in Col‐0 and *rdd* backgrounds, on which they performed fungal infection assays. Our *dme* weak‐allele mutants, but not our central cell‐specific complementation lines, displayed a strong seed‐abortion phenotype. Schumann et al. (2019) found that *DME* RNAi lines in the Col‐0 background had greater resistance against *F. oxysporum* and less suppression of defense‐related genes compared with the *rdd* mutant. In our study, both *dme* weak‐allele mutants and central cell‐specific complementation single mutants showed a hypersensitive phenotype against a bacterial pathogen and a fungal pathogen, with a stronger suppression of defense‐related gene expression compared with *rdd‐2*. These differences in phenotype between the current study and that of Schumann et al. (2019) are likely due to differences in the severity of the functional defects in DME between the two experimental systems.

DME is known to function in the central cell and the vegetative cell of the male gametophyte (Choi et al., 2002; Schoft et al., 2011; Park et al., 2017). In the vegetative nucleus of the pollen, *DME* is required for pollen germination and for the growth of the pollen tube. A complementation line expressing a *DME* native promoter‐driven DME catalytic domain sequence rescued the *dme‐2* pollen defects (Zhang et al., 2019). Surprisingly, central cell‐specific complementation lines in the current study did not show a seed‐abortion phenotype, even though the lines had a strong mutant allele background. The two promoters that were used for complementation, *DD7* and *DME* + *395*, might also allow expression in the vegetative nucleus of the pollen. Additional research will be required to determine the molecular mechanisms underlying DME‐dependent pollen growth.

An important finding of the current study is that DME is required for disease resistance against both bacterial and fungal pathogens. Although we identified several downregulated defense‐response genes and hypermethylation at their promoter regions in the *dme* mutants under normal growth conditions, we did not perform methylome and transcriptome analyses of pathogen‐infected tissues. According to our transcriptomic analysis of the uninfected plants, the basal level of the defense machinery is suppressed in the *dme* mutants.

Higher plants possess two immune systems to discriminate and defend against potential invading pathogens. One defense response is known as pathogen‐associated molecular pattern (PAMP)‐triggered immunity (PTI), and the other is termed effector‐triggered immunity (ETI) (Jones and Dangl, 2006). Pathogen‐associated molecular patterns and microbe‐associated molecular patterns (MAMPs) are recognized by plant receptors called pattern recognition receptors (PRRs), which activate the PTI response, such as a ROS burst, the production of salicylic acid, and the activation of *PR* genes (Figure S9). Meanwhile, pathogen effector proteins are recognized by resistance (*R*) genes, which are also known as NLR receptors, to activate the ETI response. Effector‐triggered immunity is often associated with programmed cell death known as the HR (Figure S9). Our results strongly indicated that DME is required for the expression of *AtPRX34*, *SOC3*, and a gene encoding a TIR‐NBS‐LRR protein. AtPRX33/34 catalyze the generation of ROS, which are important for PTI (Bindschedler et al., 2006; Daudi et al., 2012). SOC3 and the TIR‐NBS‐LRR protein play important roles in recognizing pathogen effectors to activate the ETI pathway (Monteiro and Nishimura, 2018). It appears that *DME* is required for both PTI and ETI. Mutants of the DNA methyltransferase and RNA‐directed DNA methylation pathways showed a decreased susceptibility to pathogen attack (Dowen et al., 2012; Zhang and Zhu, 2012; Deleris et al., 2016; López Sánchez et al., 2016); thus, these pathways play antagonistic roles in disease resistance (López Sánchez et al., 2016). DME may prevent the transcriptional silencing of genes important for these defense responses (Figure S9). The transcriptomic analysis of the *dme* single and *drdd* quadruple mutants suggested that DME plays an important role in regulating gene expression in vegetative tissues. The direct linkage of hyper‐DMRs in the promoter region and a downregulation of expression was only found in 205 genes, whereas more than half of the downregulated DEGs did not show hypermethylation in their promoter regions. One possibility is that DME regulates the DNA demethylation of some key regulatory genes, which in turn control the expression of these DEGs without promoter hypermethylation, such as *PR5* in the current study. Another possibility is that the regulatory elements of these DEGs lacking promoter hypermethylation are located distantly from the coding region, and that these regulatory elements may be directly targeted by DME for demethylation. The *booster1* (*b1*) locus paramutation phenotype in maize (*Zea mays*) is modulated by a hepta‐repeat sequence located 100 kbp upstream (Stam et al., 2002), so it is possible that distant regulatory sequences could be targeted by DME. It is also possible that DME and perhaps also the other three DNA demethylases may have functions in regulating gene expression independently of their activities in DNA demethylation. Future investigations will determine whether and how the plant DNA demethylases may regulate gene expression in a DNA methylation‐independent manner.

Our DNA methylome analyses of the *dme* single mutants and *drdd* quadruple mutants suggested that DME plays a role in regulating DNA demethylation in vegetative tissues, although this role is minor compared with that of ROS1. Despite this, our pathogen response assays showed that DME plays a more critical role than ROS1 in controlling pathogen resistance. DME may preferentially target the critical regulatory elements on the defense‐response genes. It is also possible that some of the effect of DME in disease resistance may not depend on its DNA demethylation activity.

## MATERIALS AND METHODS

### Gene accession numbers

*DME*, At5g04560; *ROS1*, At2g36490; *DML2*, At3g10010; *DML3*, At4g34060; *DD7*, At2g20595; *PR5*, At1g75040. SALK T‐DNA lines for *rdd‐2*: *ros1‐4*, SALK_045303; *dml2‐2*, SALK_015854; and *dml3‐2*, SALK_056440.

### Plant materials and growth conditions

*Arabidopsis thaliana* accession Col‐0 was used for all experiments. All plants were grown at 22 °C on half‐strength Murashige and Skoog (MS) medium with 1% sucrose or in soil in a 16 h light/8 h dark photoperiod.

### Plasmid construction

For the CRISPR/Cas9 constructs for *DME* mutagenesis, *AtU6* promoter‐driven single guide RNAs were constructed in a previously reported Cas9 vector (Feng et al., 2018). For complementation constructs, the *DD7* and *DME* + *395* promoters and the full‐length *DME* genomic sequence were amplified and cloned into pCambia1300. All primers used for amplifying the corresponding sequences are listed in Table S1. All transformants were generated using the flower‐dipping method.

### Whole‐genome bisulfite sequencing and data analysis

Genomic DNA was extracted from 10‐d‐old seedlings grown on half‐strength MS medium. The Genomics Core Facility at the Shanghai Center for Plant Stress Biology, China, performed the library construction, bisulfite treatment, and high‐throughput sequencing. For the data analysis, the adapters were trimmed using cutadapt (Martin, 2011), and low‐quality sequences (*q* < 20) were trimmed using Trimmomatic (Bolger et al., 2014). The clean reads were then mapped to the *Arabidopsis thaliana* TAIR 10 genome (10th release of the *Arabidopsis thaliana* genome sequence from the Arabidopsis Information Resource) using the Bisulfite Sequence Mapping Program (BSMAP) (Xi and Li, 2009) with a 0.08 mismatch rate. The methratio.py script was used to determine the methylation ratio from the BSMAP mapping results, with the option – *r* used to remove potential PCR duplicates, and – *z* used to report loci with zero methylation ratios.

### Differential methylation analysis

Differentially methylated regions were identified as previously described with minor modifications (Zhang et al., 2013). In brief, only cytosines with a depth of at least four in the library were considered for further analysis. A sliding‐window approach with a 200 bp window and a 50 bp step size was used to identify DMRs. Fisher's exact test was used to compare the methylated and unmethylated cytosines within each window, with a *P*‐value cutoff of 0.05. False discovery rates (FDRs) were then estimated using a Benjamini–Hochberg adjustment of Fisher's *P*‐value in the R environment. Windows with an FDR < 0.01 and a fold change >1.5 in the DNA methylation level and with at least five DMCs (defined as a dmC with *P* < 0.01 in Fisher's exact test) were used for further analysis. Windows within 100 bp of each other were merged into a larger region, which was then adjusted to shrink to the first and last DMC.

### Whole‐genome transcriptomic analysis

Total RNA was isolated from 10‐d‐old seedlings using RNeasy Plant (Qiagen, Hilden, Germany). Transcriptome libraries were prepared from extracted total RNA samples by the Genomics Core Facility at the Shanghai Center for Plant Stress Biology. Adapters and low‐quality sequences (*q* < 20) were trimmed using cutadapt (Martin, 2011) and Trimmomatic (Bolger et al., 2014), respectively, and the trimmed reads were aligned to the TAIR 10 genome using STAR (Dobin et al., 2013). The tool htseq‐count from the Python package HTSeq (Anders et al., 2015) was used to count the mapped fragments for each gene. The output count table was used as the input for DESeq (Anders and Huber, 2010) to compute the DEGs between pairs of samples, according to a FDR threshold of <0.01 and a fold change value of ≥1.5. Differentially methylated regions‐related genes were defined as genes with DMRs within their 2 kb promoter regions.

### Bacterial infection assay with syringe inoculation

Plant resistance against the bacterial pathogen *Pst* DC3000 was measured as described previously (Li et al., 2015; Yang et al., 2015). *Pst* DC3000 was grown at 28°C on lysogeny broth (LB) solid medium with 50 mg/L rifampicin. After 2 d, single colonies were transferred to liquid LB medium supplemented with 50 mg/L rifampicin at 28°C. *Arabidopsis* seedlings were grown under short‐day conditions (8 h light/16 h dark photoperiod) until they bolted. A syringe was used to inoculate the fully opened leaves of 4‐week‐old plants with a *Pst* DC3000 suspension at an optical density of 0.002 at 600 nm. For the mock treatment, leaves were inoculated with 10 mmol/L MgCl_2_. Disks from the inoculated areas of the leaves were collected at 0 and 4 d to determine the number of colony‐forming units (CFUs) per unit of leaf tissue, as described in the “Assessment of bacterial biomass” section.

### Bacterial infection assay on plates

*Pst DC3000* was prepared as described in the previous section. The bacterial suspension was diluted with sterile distilled water to an optical density of 0.02 at 600 nm, then Silwet L‐77 was added to a final concentration of 0.025%. A 50 mL volume of the bacterial suspension was added to a plate on which an *Arabidopsis* seedling had grown for 2 weeks (Ishiga et al., 2011; Liu et al., 2015). After a 3 min incubation, the bacterial suspension was poured off the plates, which were returned to the growth chamber. The bacterial biomass was detected at 0–4 dpi, as described in the next section.

### Assessment of bacterial biomass

The leaves of the inoculated and control plants were collected, weighed, surface‐sterilized in 5% H_2_O_2_ for 3 min, and then rinsed with sterile distilled water. The leaves of each replicate were homogenized, and 1 mL of sterile distilled water was added to the homogenate. The suspension was diluted to the proper concentration and plated on LB medium containing rifampicin (50 mg/L). After 4 d, the colonies were counted and expressed as CFUs/mg of tissue.

### Fungal infection assay

*Verticillium dahlia* strain V592 (Broekaert et al., 1990; Ellendorff et al., 2009) was grown on potato dextrose agar at 28 °C for 2 d before several colonies were transferred to Czapek–Dox liquid medium at 28 °C. A spore suspension of *V. dahlia* in Czapek–Dox liquid medium was obtained. The roots of 2‐week‐old plate‐grown *Arabidopsis* seedlings were dipped for 5 min into the *V. dahlia* spore suspension or into Czapek–Dox liquid medium as a mock treatment. The seedlings were then transplanted into soil and the trays were covered with clear lids to maintain the humidity for the experiments. At 25–30 d after inoculation (at which time the symptoms were visible), the leaves were collected for the assessment of fungal biomass and the plant defense gene expression levels. DNA extraction for *V. dahlia* biomass assessment by qPCR with fungus‐specific ITS1‐F primer (AAAGTTTTAATGGTTCGCTAAGA) in combination with the *V. dahlia*‐special reverse primer (CTTGGTCATTTAGAGGAAGTAA). And for normalization, we used RuBisCO, the *Arabidopsis* large subunit, for primer set.

## AUTHOR CONTRIBUTIONS

D.M., W.J.Z., and J.K.Z. designed the research; W.J.Z. and D.M. performed the experiments with support from X.Q.L., C.Z., K.I.K., C.F.H., and C.G.D.; H.H. performed the informatics analysis; D.M. and J.K.Z. supervised the project; D.M., W.J.Z., H.H., H.M.Z., and J.K.Z. wrote the manuscript. All authors read and approved of its content.

## Supporting information

Additional Supporting Information may be found online in the supporting information tab for this article: http://onlinelibrary.wiley.com/doi/10.1111/jipb.13037/suppinfo

**Figure S1.** Expression of the 5‐methylcytosine DNA glycosylase family genes
(**A**) Expression of the 5‐methylcytosine DNA glycosylase genes in different tissues. The data were obtained from the public *Arabidopsis* transcriptome database AtGenExpress. (**B**) Expression of the 5‐methylcytosine DNA glycosylase genes in seedlings of Col‐0 and the *ros1‐4*, *rdd‐2*, *met1‐3*, *drm2 drm3 cmt3* (*ddc*), and *ddm1* mutants as determined using quantitative real‐time polymerase chain reaction (qRT‐PCR). Data shown are means ± *SEM* from three experiments.
**Figure S2.** Expression of the 5‐methylcytosine DNA glycosylase genes in the weak *dme* mutants
(**A**) *ROS1* expression. (**B**) *DML2* expression. (**C**) *DML3* expression. Data shown are means ± *SEM* from three experiments.
**Figure S3.** Characterization of the methylation of the *dme* single mutants
(**A**) Individual boxplot analysis of the DNA methylation levels (relative to Col‐0) of *dme‐A‐Del*, *dme‐T‐In*, *dme‐3‐In*, and *dme* ^*DD7 pro*^ mutant‐specific hyper‐differentially methylated regions (hyper‐DMRs). Methylation levels of mC, mCG, mCHG, and mCHH contexts are shown for Col‐0, *rdd‐2*, and four *dme* weak‐allele mutants with replicates (with the same color) (*P < 10^−5^ compared with Col‐0, one‐tailed Wilcoxon tests). (**B**) Heatmap analysis of the DNA methylation level of the hyper‐DMRs of the *dme* single mutants in Col‐0, *rdd‐2*, and *dme* single mutants with biological repeats.
**Figure S4.** Characterization of the central cell‐specific complementation *drdd* quadruple mutants
(**A**) Box plots of hyper‐differentially methylated regions (hyper‐DMRs) specific to the *drdd* ^*+395 pro*^ quadruple mutant. The mC, mCG, mCHG, and mCHH contexts are shown for Col‐0, *rdd‐2*, *drdd* ^*+395 pro*^, and *drdd* ^*DD7 pro*^ mutants with replicates in the same color. The analysis was performed relative to Col‐0 (*P < 10^−8^ compared with Col‐0, one‐tailed Wilcoxon tests). (**B**) Heatmap analysis of the DNA methylation levels of the hyper‐DMRs of the central cell‐specific complementation quadruple mutants in Col‐0, *rdd‐2*, and *drdd* mutants. (**C**) Boxplot analysis of the distance from the gene start site to the transposable elements (TEs) targeted (hypermethylated) and not targeted (not hypermethylated) in *rdd‐2*, *dme* ^*DD7 pro*^, *drdd* ^*+395 pro*^, and *drdd* ^*DD7 pro*^ (*P < 10^−5^ compared with Col‐0, one‐tailed Wilcoxon tests).
**Figure S5.** Transcriptome analysis of the *drdd* quadruple mutants
(**A**) Overlap of downregulated differentially expressed genes (DEGs) among *rdd‐2, drdd* ^*+395 pro*^, and *drdd* ^*DD7 pro*^. (**B**) Heatmap analysis of the specific downregulated DEGs in *rdd‐2*, *dme* ^*DD7 pro*^, *drdd* ^*+395 pro*^, and *drdd* ^*DD7 pro*^.
**Figure S6.** *Pst* DC3000 infection phenotype in the *dme* weak‐allele mutants
(**A**) Leaf symptoms of *dme* weak‐allele mutants that were syringe‐inoculated with *Pst* DC3000 at 4 d post‐inoculation (dpi). (**B**) Symptoms of *drdd* quadruple mutants that were exposed to *Pst* DC3000 on agar plates. (**C**) Bacterial biomass in *dme* weak‐allele mutants at 4 dpi. Data shown are means ± *SEM* from three experiments (*P < 0.05, **P < 0.01, one‐tailed Student's *t*‐test). (**D**) Expression of *PR5* in *dme* weak‐allele‐mutants at 4 dpi, as determined using quantitative real‐time polymerase chain reaction (qRT‐PCR). Data shown are means ± *SEM* from three experiments (**P < 0.01, one‐tailed Student's *t*‐test). (**E**,**F**) Expression of *DME* (E) and *ROS1* (F) in Col‐0 at 3, 6, and 9 h after the flg22 treatment, as determined using qRT‐PCR (NS; not significant, one‐tailed Student's *t*‐test).
**Figure S7.** *Verticillium dahliae* infection phenotype in the *dme* weak‐allele mutants
(**A**) Symptoms of *V. dahlia*‐inoculated *dme* weak‐allele mutants at 28 d post‐inoculation (dpi). (**B**) *V. dahliae* biomass in the leaves of inoculated *dme* weak‐allele mutants at 28 dpi. Data shown are means ± *SEM* from three experiments (**P < 0.01, one‐tailed Student's *t*‐test). (**C**) Expression of *PR5* in inoculated *dme* weak‐allele mutants, as determined using a quantitative real‐time polymerase chain reaction (qRT‐PCR). Data shown are means ± *SEM* from three experiments (**P < 0.01, one‐tailed Student's *t*‐test).
**Figure S8.** Expression analysis of defense‐related genes in *Pst* DC3000–infected *dme* weak‐allele mutants, with Col‐0 as a control
Data shown are means ± *SEM* from three experiments. (**A**) Expression of At1g72890, a TIR‐NBS‐LRR class disease resistance protein. (**B**) Expression of At2g02100, *PDF1.2*. (**C**) Expression of At3g49120, the class III peroxidase *PERX34* (**P < 0.01, one‐tailed Student's *t*‐test).
**Figure S9.** A working model of DME function in the disease resistance response
Pathogen attack activates the pathogen‐associated molecular pattern (PAMP)‐triggered immunity (PTI) and effector‐triggered immunity (ETI) defense responses in *Arabidopsis*. Pattern recognition receptors (PRRs), which are cell surface receptors, recognize the PAMPs or microbe‐associated molecular patterns (MAMPs) to activate PTI. The PTI response pathway includes AtPRX33/34‐dependent and ‐independent reactive oxygen species (ROS) production, followed by salicylic acid biosynthesis and pathogenesis‐related (*PR*) gene expression. Pathogens can deliver effector proteins into host cells to suppress the PTI. As a counter measure, the effector proteins can be recognized by the intracellular receptors (R proteins), which in turn can activate the ETI response to induce the hypersensitive response (HR) cell death. *DME* is required for the expression of *AtPRX34*, *SOC3*, and a TIR‐NBS‐LRR‐encoding gene, which encode key components of the PTI and ETI response pathways.

## References

[jipb13037-bib-0001] Anders, S. , and Huber, W. (2010). Differential expression analysis for sequence count data. Genome Biol. 11: R106. 20979621 10.1186/gb-2010-11-10-r106 PMC3218662

[jipb13037-bib-0002] Anders, S. , Pyl, P.T. , and Huber, W. (2015). HTSeq‐‐a Python framework to work with high‐throughput sequencing data. Bioinformatics 31: 166–169. 25260700 10.1093/bioinformatics/btu638 PMC4287950

[jipb13037-bib-0003] Bindschedler, L.V. , Dewdney, J. , Blee, K.A. , Stone, J.M. , Asai, T. , Plotnikov, J. , Denoux, C. , Hayes, T. , Gerrish, C. , Davies, D.R. , Ausubel, F.M. , and Bolwell, G.P. (2006). Peroxidase‐dependent apoplastic oxidative burst in *Arabidopsis* required for pathogen resistance. Plant J. 47: 851–863. 16889645 10.1111/j.1365-313X.2006.02837.x PMC3233234

[jipb13037-bib-0004] Bolger, A.M. , Lohse, M. , and Usadel, B. (2014). Trimmomatic: a flexible trimmer for Illumina sequence data. Bioinformatics 30: 2114–2120. 24695404 10.1093/bioinformatics/btu170 PMC4103590

[jipb13037-bib-0005] Broekaert, W.F. , Terras, F.R.G. , Cammue, B.P.A. , and Vanderleyden, J. (1990). An automated quantitative assay for fungal growth inhibition. FEMS Microbiol. Lett. 69: 55–59.

[jipb13037-bib-0006] Choi, Y. , Gehring, M. , Johnson, L. , Hannon, M. , Harada, J.J. , Goldberg, R.B. , Jacobsen, S.E. , and Fischer, R.L. (2002). DEMETER, a DNA glycosylase domain protein, is required for endosperm gene imprinting and seed viability in *Arabidopsis* . Cell 110: 33–42. 12150995 10.1016/s0092-8674(02)00807-3

[jipb13037-bib-0007] Daudi, A. , Cheng, Z. , O’Brien, J.A. , Mammarella, N. , Khan, S. , Ausubel, F.M. , and Bolwell, G.P. (2012). The apoplastic oxidative burst peroxidase in *Arabidopsis* is a major component of Pattern‐triggered immunity. Plant Cell 24: 275. 22247251 10.1105/tpc.111.093039 PMC3289579

[jipb13037-bib-0008] Deleris, A. , Halter, T. , and Navarro, L. (2016). DNA methylation and demethylation in plant immunity. Annu. Rev. Phytopathol. 54: 579–603. 27491436 10.1146/annurev-phyto-080615-100308

[jipb13037-bib-0009] Dobin, A. , Davis, C.A. , Schlesinger, F. , Drenkow, J. , Zaleski, C. , Jha, S. , Batut, P. , Chaisson, M. , and Gingeras, T.R. (2013). STAR: Ultrafast universal RNA‐seq aligner. Bioinformatics 29: 15–21. 23104886 10.1093/bioinformatics/bts635 PMC3530905

[jipb13037-bib-0010] Dowen, R.H. , Pelizzola, M. , Schmitz, R.J. , Lister, R. , Dowen, J.M. , Nery, J.R. , Dixon, J.E. , and Ecker, J.R. (2012). Widespread dynamic DNA methylation in response to biotic stress. Proc. Natl. Acad. Sci. USA 109: E2183–2191. 22733782 10.1073/pnas.1209329109 PMC3420206

[jipb13037-bib-0011] Ellendorff, U. , Fradin, E.F. , de Jonge, R. , and Thomma, B.P.H.J. (2009). RNA silencing is required for *Arabidopsis* defence against Verticillium wilt disease. J. Exp. Bot. 60: 591–602. 19098131 10.1093/jxb/ern306 PMC2651451

[jipb13037-bib-0012] Feng, Z. , Zhang, Z. , Hua, K. , Gao, X. , Mao, Y. , Botella, J. , and Zhu, J.‐K. (2018). A highly efficient cell Division‐specific CRISPR/Cas9 system generates homozygous mutants for multiple genes in *Arabidopsis* . Int. J. Mol. Sci. 19: 3925. 10.3390/ijms19123925 PMC6321140 30544514

[jipb13037-bib-0013] Gehring, M. , Huh, J.H. , Hsieh, T.F. , Penterman, J. , Choi, Y. , Harada, J.J. , Goldberg, R.B. , and Fischer, R.L. (2006). DEMETER DNA glycosylase establishes MEDEA polycomb gene self‐imprinting by allele‐specific demethylation. Cell 124: 495–506. 16469697 10.1016/j.cell.2005.12.034 PMC4106368

[jipb13037-bib-0014] Huettel, B. , Kanno, T. , Daxinger, L. , Bucher, E. , van der Winden, J. , Matzke, A.J.M. , and Matzke, M. (2007). RNA‐directed DNA methylation mediated by DRD1 and Pol IVb: A versatile pathway for transcriptional gene silencing in plants. Biochim. Biophys. Acta 1769: 358–374. 17449119 10.1016/j.bbaexp.2007.03.001

[jipb13037-bib-0015] Ishiga, Y. , Ishiga, T. , Uppalapati, S.R. , and Mysore, K.S. (2011). *Arabidopsis* seedling flood‐inoculation technique: a rapid and reliable assay for studying plant‐bacterial interactions. Plant Methods 7: 32. 21978451 10.1186/1746-4811-7-32 PMC3206466

[jipb13037-bib-0016] Jones, J.D.G. , and Dangl, J.L. (2006). The plant immune system. Nature 444: 323–329. 17108957 10.1038/nature05286

[jipb13037-bib-0017] Klepikova, A.V. , Kasianov, A.S. , Gerasimov, E.S. , Logacheva, M.D. , and Penin, A.A. (2016). A high resolution map of the *Arabidopsis thaliana* developmental transcriptome based on RNA‐seq profiling. Plant J. 88: 1058–1070. 27549386 10.1111/tpj.13312

[jipb13037-bib-0018] Le, T. , Schumann, U. , Smith, N.A. , Tiwari, S. , Au, P.C.K. , Zhu, Q. , Taylor, J.M. , Kazan, K. , Llewellyn, D.J. , Zhang, R. , Dennis, E.S. , and Wang, M. (2014). DNA demethylases target promoter transposable elements to positively regulate stress responsive genes in *Arabidopsis* . Genome Bio. 15: 458. 25228471 10.1186/s13059-014-0458-3 PMC4189188

[jipb13037-bib-0019] Lei, M. , Zhang, H. , Julian, R. , Tang, K. , Xie, S. , and Zhu, J.‐K. (2015). Regulatory link between DNA methylation and active demethylation in *Arabidopsis* . Proc. Natl. Acad. Sci. USA 112: 3553. 25733903 10.1073/pnas.1502279112 PMC4371987

[jipb13037-bib-0020] Li, X. , Sun, Z. , Shao, S. , Zhang, S. , Ahammed, G.J. , Zhang, G. , Jiang, Y. , Zhou, J. , Xia, X. , Zhou, Y. , Yu, J. , and Shi, K. (2015). Tomato‐Pseudomonas syringae interactions under elevated CO₂ concentration: the role of stomata. J. Exp. Bot. 66: 307–316. 25336683 10.1093/jxb/eru420 PMC4265165

[jipb13037-bib-0021] Lister, R. , O'Malley, R.C. , Tonti‐Filippini, J. , Gregory, B.D. , Berry, C.C. , Millar, A.H. , and Ecker, J.R. (2008). Highly integrated Single‐base resolution maps of the epigenome in *Arabidopsis* . Cell 133: 523–536. 18423832 10.1016/j.cell.2008.03.029 PMC2723732

[jipb13037-bib-0022] Liu, R. , and Lang, Z. (2020). The mechanism and function of active DNA demethylation in plants. J. Integr. Plant Biol. 62: 148–159. 31628716 10.1111/jipb.12879

[jipb13037-bib-0023] Liu, X. , Sun, Y. , Kørner, C.J. , Du, X. , Vollmer, M.E. , and Pajerowska‐Mukhtar, K.M. (2015). Bacterial leaf infiltration assay for fine characterization of plant defense responses using the *Arabidopsis thaliana‐Pseudomonas* syringae pathosystem. JoVE e53364. 10.3791/53364 PMC4692633 26485301

[jipb13037-bib-0024] López Sánchez, A. , Stassen, J.H.M. , Furci, L. , Smith, L.M. , and Ton, J. (2016). The role of DNA (de)methylation in immune responsiveness of *Arabidopsis* . Plant J. 88: 361–374. 27341062 10.1111/tpj.13252 PMC5132069

[jipb13037-bib-0025] Luo, C. , Sidote, D.J. , Zhang, Y. , Kerstetter, R.A. , Michael, T.P. , and Lam, E. (2013). Integrative analysis of chromatin states in *Arabidopsis* identified potential regulatory mechanisms for natural antisense transcript production. Plant J. 73: 77–90. 22962860 10.1111/tpj.12017

[jipb13037-bib-0026] Martin, M. (2011). Cutadapt removes adapter sequences from high throughput sequencing reads. EMBnet J. 17: 10–12.

[jipb13037-bib-0027] Martínez‐Macías, M.aríaI. , Qian, W. , Miki, D. , Pontes, O. , Liu, Y. , Tang, K. , Liu, R. , Morales‐Ruiz, T. , Ariza , Rafael, R. , Roldán‐Arjona, T. , and Zhu, J.‐K. (2012). A DNA 3′ Phosphatase functions in active DNA demethylation in *Arabidopsis* . Mol. Cell 45: 357–370. 22325353 10.1016/j.molcel.2011.11.034 PMC3278721

[jipb13037-bib-0028] Mathieu, O. , Reinders, J. , Čaikovski, M. , Smathajitt, C. , and Paszkowski, J. (2007). Transgenerational stability of the *Arabidopsis* epigenome is coordinated by CG methylation. Cell 130: 851–862. 17803908 10.1016/j.cell.2007.07.007

[jipb13037-bib-0029] Monteiro, F. , and Nishimura, M.T. (2018). Structural, functional, and genomic diversity of plant NLR proteins: An evolved resource for rational engineering of plant immunity. Annu. Rev. Phytopathol. 56: 243–267. 29949721 10.1146/annurev-phyto-080417-045817

[jipb13037-bib-0030] Park, J.‐S. , Frost, J.M. , Park, K. , Ohr, H. , Park, G.T. , Kim, S. , Eom, H. , Lee, I. , Brooks, J.S. , Fischer, R.L. , and Choi, Y. (2017). Control of DEMETER DNA demethylase gene transcription in male and female gamete companion cells in *Arabidopsis thaliana* . Proc. Natl. Acad. Sci. USA 114: 2078. 28130550 10.1073/pnas.1620592114 PMC5338364

[jipb13037-bib-0031] Penterman, J. , Zilberman, D. , Huh, J.H. , Ballinger, T. , Henikoff, S. , and Fischer, R.L. (2007). DNA demethylation in the *Arabidopsis* genome. Proc. Natl. Acad. Sci. USA 104: 6752. 17409185 10.1073/pnas.0701861104 PMC1847597

[jipb13037-bib-0032] Schmid, M. , Davison, T.S. , Henz, S.R. , Pape, U.J. , Demar, M. , Vingron, M. , Schölkopf, B. , Weigel, D. , and Lohmann, J.U. (2005). A gene expression map of *Arabidopsis thaliana* development. Nat. Genet. 37: 501–506. 15806101 10.1038/ng1543

[jipb13037-bib-0033] Schoft, V.K. , Chumak, N. , Choi, Y. , Hannon, M. , Garcia‐Aguilar, M. , Machlicova, A. , Slusarz, L. , Mosiolek, M. , Park, J.S. , Park, G.T. , Fischer, R.L. , and Tamaru, H. (2011). Function of the DEMETER DNA glycosylase in the *Arabidopsis thaliana* male gametophyte. Proc. Natl. Acad. Sci. USA 108: 8042–8047. 21518889 10.1073/pnas.1105117108 PMC3093457

[jipb13037-bib-0034] Schumann, U. , Lee, J. , Kazan, K. , Ayliffe, M. , and Wang, M.B. (2017). DNA‐Demethylase Regulated genes show Methylation‐independent spatiotemporal expression patterns. Front. Plant Sci. 8: 1449–1449. 28894455 10.3389/fpls.2017.01449 PMC5581395

[jipb13037-bib-0035] Schumann, U. , Lee, J.M. , Smith, N.A. , Zhong, C. , Zhu, J.K. , Dennis, E.S. , Millar, A.A. , and Wang, M.B. (2019). DEMETER plays a role in DNA demethylation and disease response in somatic tissues of *Arabidopsis* . Epigenetics 14: 1074–1087. 31189415 10.1080/15592294.2019.1631113 PMC6773409

[jipb13037-bib-0036] Stam, M. , Belele, C. , Dorweiler, J.E. , and Chandler, V.L. (2002). Differential chromatin structure within a tandem array 100 kb upstream of the maize b1 locus is associated with paramutation. Genes. Dev. 16: 1906–1918. 12154122 10.1101/gad.1006702 PMC186425

[jipb13037-bib-0037] Steffen, J.G. , Kang, I.‐H. , Macfarlane, J. , and Drews, G.N. (2007). Identification of genes expressed in the *Arabidopsis* female gametophyte. Plant J. 51: 281–292. 17559508 10.1111/j.1365-313X.2007.03137.x

[jipb13037-bib-0038] Stroud, H. , Greenberg, M.V.C. , Feng, S. , Bernatavichute, Y.V. , and Jacobsen, S.E. (2013). Comprehensive analysis of silencing mutants reveals complex regulation of the *Arabidopsis* methylome. Cell 152: 352–364. 23313553 10.1016/j.cell.2012.10.054 PMC3597350

[jipb13037-bib-0039] Tang, K. , Lang, Z. , Zhang, H. , and Zhu, J.K. (2016). The DNA demethylase ROS1 targets genomic regions with distinct chromatin modifications. Nat. Plants 2: 16169. 27797352 10.1038/nplants.2016.169 PMC5123759

[jipb13037-bib-0040] Tsuzuki, M. , Takeda, A. , and Watanabe, Y. (2014). Recovery of dicer‐like 1‐late flowering phenotype by miR172 expressed by the noncanonical DCL4‐dependent biogenesis pathway. RNA 20: 1320–1327. 24966167 10.1261/rna.044966.114 PMC4105755

[jipb13037-bib-0041] Xi, Y. , and Li, W. (2009). BSMAP: whole genome bisulfite sequence MAPping program. BMC Bioinformatics 10: 232. 19635165 10.1186/1471-2105-10-232 PMC2724425

[jipb13037-bib-0042] Yamamuro, C. , Miki, D. , Zheng, Z. , Ma, J. , Wang, J. , Yang, Z. , Dong, J. , and Zhu, J.‐K. (2014). Overproduction of stomatal lineage cells in *Arabidopsis* mutants defective in active DNA demethylation. Nat. Commun. 5: 4062. 24898766 10.1038/ncomms5062 PMC4097119

[jipb13037-bib-0043] Yang, D.‐L. , Zhang, G. , Tang, K. , Li, J. , Yang, L. , Huang, H. , Zhang, H. , and Zhu, J.‐K. (2016). Dicer‐independent RNA‐directed DNA methylation in *Arabidopsis* . Cell Res. 26: 66. 26642813 10.1038/cr.2015.145 PMC4816133

[jipb13037-bib-0044] Yang, L. , Li, B. , Zheng, X.Y. , Li, J. , Yang, M. , Dong, X. , He, G. , An, C. , and Deng, X.W. (2015). Salicylic acid biosynthesis is enhanced and contributes to increased biotrophic pathogen resistance in *Arabidopsis* hybrids. Nat. Commun. 6: 7309. 26065719 10.1038/ncomms8309 PMC4490401

[jipb13037-bib-0045] Yu, A. , Lepère, G. , Jay, F. , Wang, J. , Bapaume, L. , Wang, Y. , Abraham, A.‐L. , Penterman, J. , Fischer, R.L. , Voinnet, O. , and Navarro, L. (2013). Dynamics and biological relevance of DNA demethylation in *Arabidopsis* antibacterial defense. Proc. Natl. Acad. Sci. USA 110: 2389. 23335630 10.1073/pnas.1211757110 PMC3568381

[jipb13037-bib-0046] Zhang, C. , Hung, Y.H. , Rim, H.J. , Zhang, D. , Frost, J.M. , Shin, H. , Jang, H. , Liu, F. , Xiao, W. , Iyer, L.M. , Aravind, L. , Zhang, X.Q. , Fischer, R.L. , Huh, J.H. , and Hsieh, T.‐F. (2019). The catalytic core of DEMETER guides active DNA demethylation in *Arabidopsis* . Proc. Natl. Acad. Sci. USA 116: 17563–17571. 31409710 10.1073/pnas.1907290116 PMC6717269

[jipb13037-bib-0047] Zhang, H. , Lang, Z. , and Zhu, J.K. (2018). Dynamics and function of DNA methylation in plants. Nature Reviews Molecular Cell Biology 19: 489–506. 29784956 10.1038/s41580-018-0016-z

[jipb13037-bib-0048] Zhang, H. , Ma, Z.Y. , Zeng, L. , Tanaka, K. , Zhang, C.J. , Ma, J. , Bai, G. , Wang, P. , Zhang, S.W. , Liu, Z.W. , Cai, T. , Tang, K. , Liu, R. , Shi, X. , He, X.J. , and Zhu, J.K. (2013). DTF1 is a core component of RNA‐directed DNA methylation and may assist in the recruitment of Pol IV. Proc. Natl. Acad. Sci. USA 110: 8290. 23637343 10.1073/pnas.1300585110 PMC3657815

[jipb13037-bib-0049] Zhang, H. , and Zhu, J.K. (2012). Active DNA demethylation in plants and animals. Cold Spring Harbor Symp. Quant. Biol. 77: 161–173. 23197304 10.1101/sqb.2012.77.014936 PMC3657592

[jipb13037-bib-0050] Zhu, J.K. (2009). Active DNA Demethylation Mediated by DNA Glycosylases. Annu. Rev. Genet. 43: 143–166. 19659441 10.1146/annurev-genet-102108-134205 PMC3137514

[jipb13037-bib-0051] Zhu, J. , Kapoor, A. , Sridhar, V.V. , Agius, F. , and Zhu, J.K. (2007). The DNA Glycosylase/Lyase ROS1 functions in pruning DNA methylation patterns in *Arabidopsis* . Curr. Biol. 17: 54–59. 17208187 10.1016/j.cub.2006.10.059

