# Supplementary material for: Roles of DEMETER in regulating DNA methylation in vegetative tissues and pathogen resistance
Source: J Integr Plant Biol. 2021 Mar 16;63(4):691–706. doi: 10.1111/jipb.13037 (PMC8251943; doi:10.1111/jipb.13037)
Supplement: Supplementary file 1 — Figure S1. Expression of the 5‐methylcytosine DNA glycosylase family genes (A) Expression of the 5‐methylcytosine DNA glycosylase genes in different tissues. The data were obtained from the public Arabidopsis transcriptome database AtGenExpress. (B) Expression of the 5‐methylcytosine DNA glycosylase genes in seedlings of Col‐0 and the ros1‐4, rdd‐2, met1‐3, drm2 drm3 cmt3 (ddc), and ddm1 mutants as determined using quantitative real‐time polymerase chain reaction (qRT‐PCR). Data shown are means ± SEM from three experiments. Figure S2. Expression of the 5‐methylcytosine DNA glycosylase genes in the weak dme mutants (A) ROS1 expression. (B) DML2 expression. (C) DML3 expression. Data shown are means ± SEM from three experiments. Figure S3. Characterization of the methylation of the dme single mutants (A) Individual boxplot analysis of the DNA methylation levels (relative to Col‐0) of dme‐A‐Del, dme‐T‐In, dme‐3‐In, and dme DD7 pro mutant‐specific hyper‐differentially methylated regions (hyper‐DMRs). Methylation levels of mC, mCG, mCHG, and mCHH contexts are shown for Col‐0, rdd‐2, and four dme weak‐allele mutants with replicates (with the same color) (*P < 10−5 compared with Col‐0, one‐tailed Wilcoxon tests). (B) Heatmap analysis of the DNA methylation level of the hyper‐DMRs of the dme single mutants in Col‐0, rdd‐2, and dme single mutants with biological repeats. Figure S4. Characterization of the central cell‐specific complementation drdd quadruple mutants (A) Box plots of hyper‐differentially methylated regions (hyper‐DMRs) specific to the drdd +395 pro quadruple mutant. The mC, mCG, mCHG, and mCHH contexts are shown for Col‐0, rdd‐2, drdd +395 pro, and drdd DD7 pro mutants with replicates in the same color. The analysis was performed relative to Col‐0 (*P < 10−8 compared with Col‐0, one‐tailed Wilcoxon tests). (B) Heatmap analysis of the DNA methylation levels of the hyper‐DMRs of the central cell‐specific complementation quadruple mutants in Col‐0, rdd‐2, and drdd [file JIPB-63-691-s001.pdf]

A

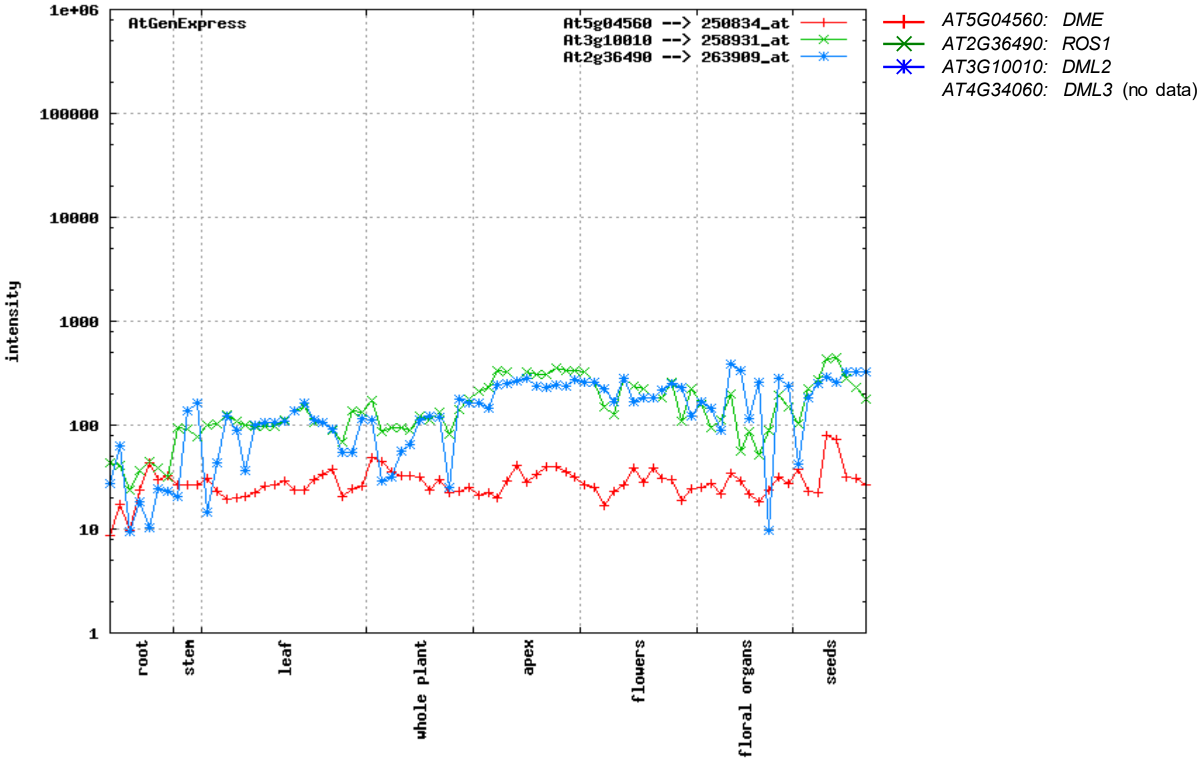

B

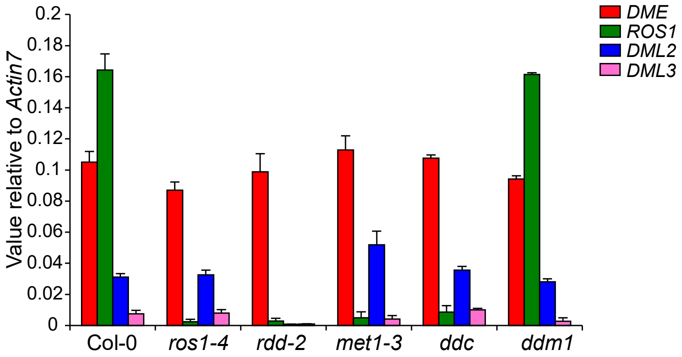

Supplementary Figure 1. Expression of the 5-methylcytosine DNA glycosylase family genes.

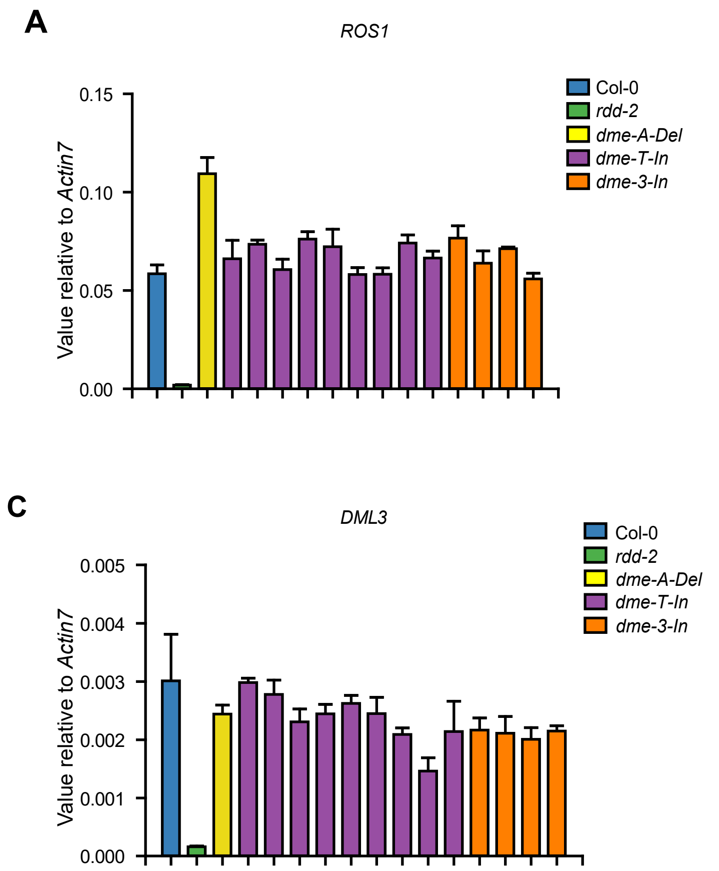

Supplementary Figure 2. Expression of the 5-methylcytosine DNA glycosylase genes in the dme weak-allele mutants.

A

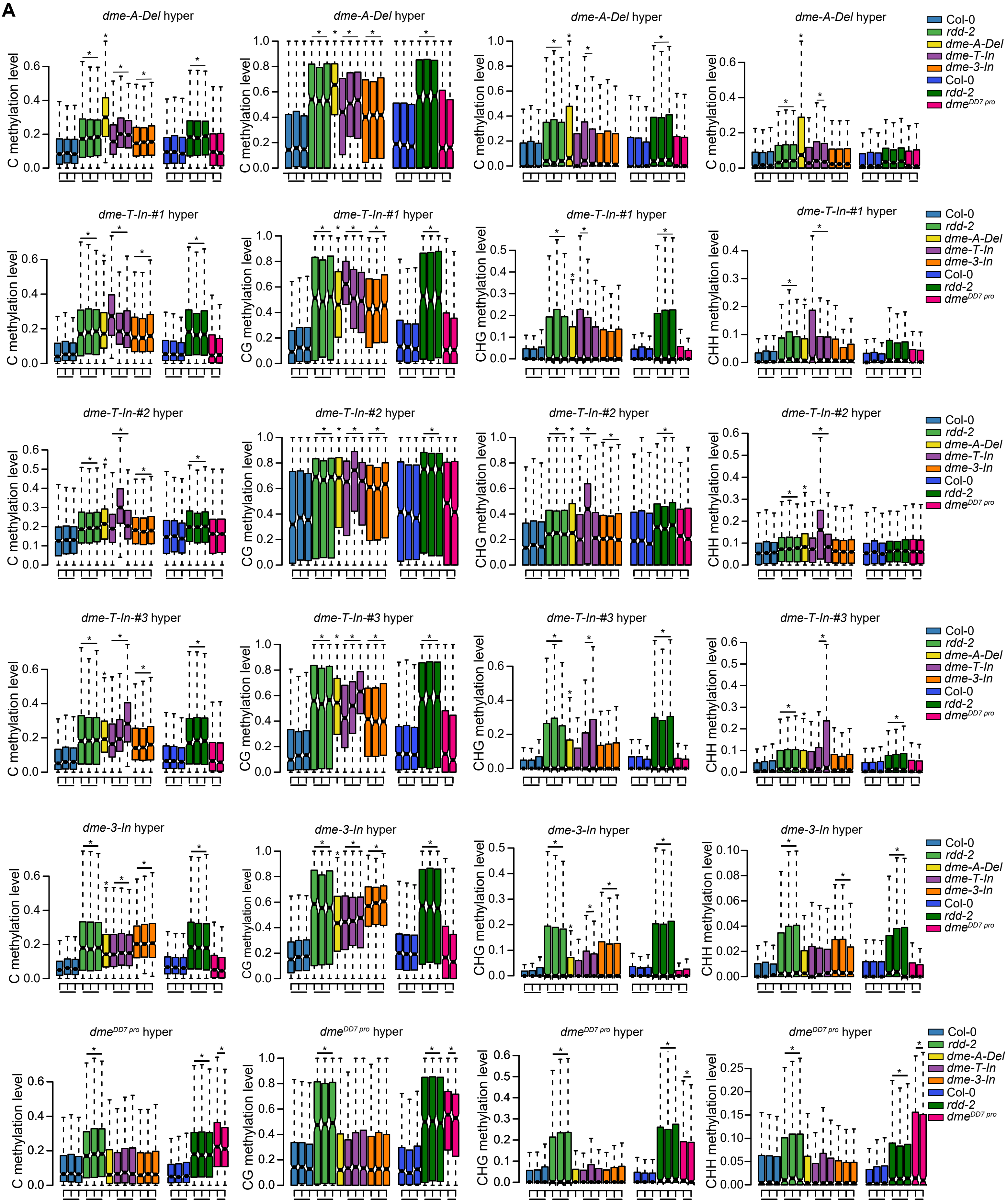

dme-A-Del hyper
dme-T-In-#1 hyper
dme-T-In-#2 hyper
dme-T-In-#3 hyper
dme-3-In hyper
dme^DD7 pro hyper
C methylation level
CG methylation level
CHG methylation level
CHH methylation level
Col-0
rdd-2
dme-A-Del
dme-T-In
dme-3-In
Col-0
rdd-2
dme^DD7 pro

B

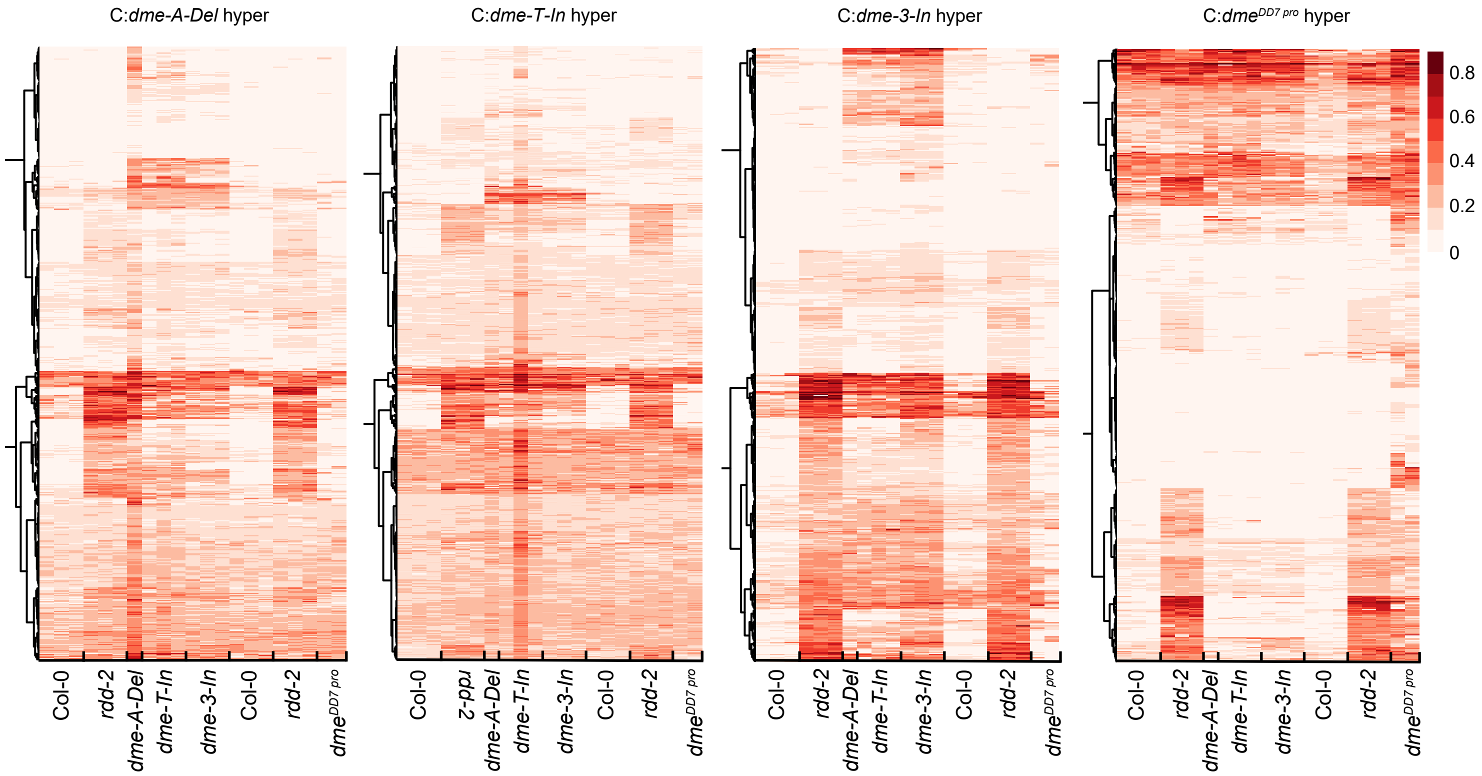

Supplementary Figure 3. Characterization of *dme* single mutants.

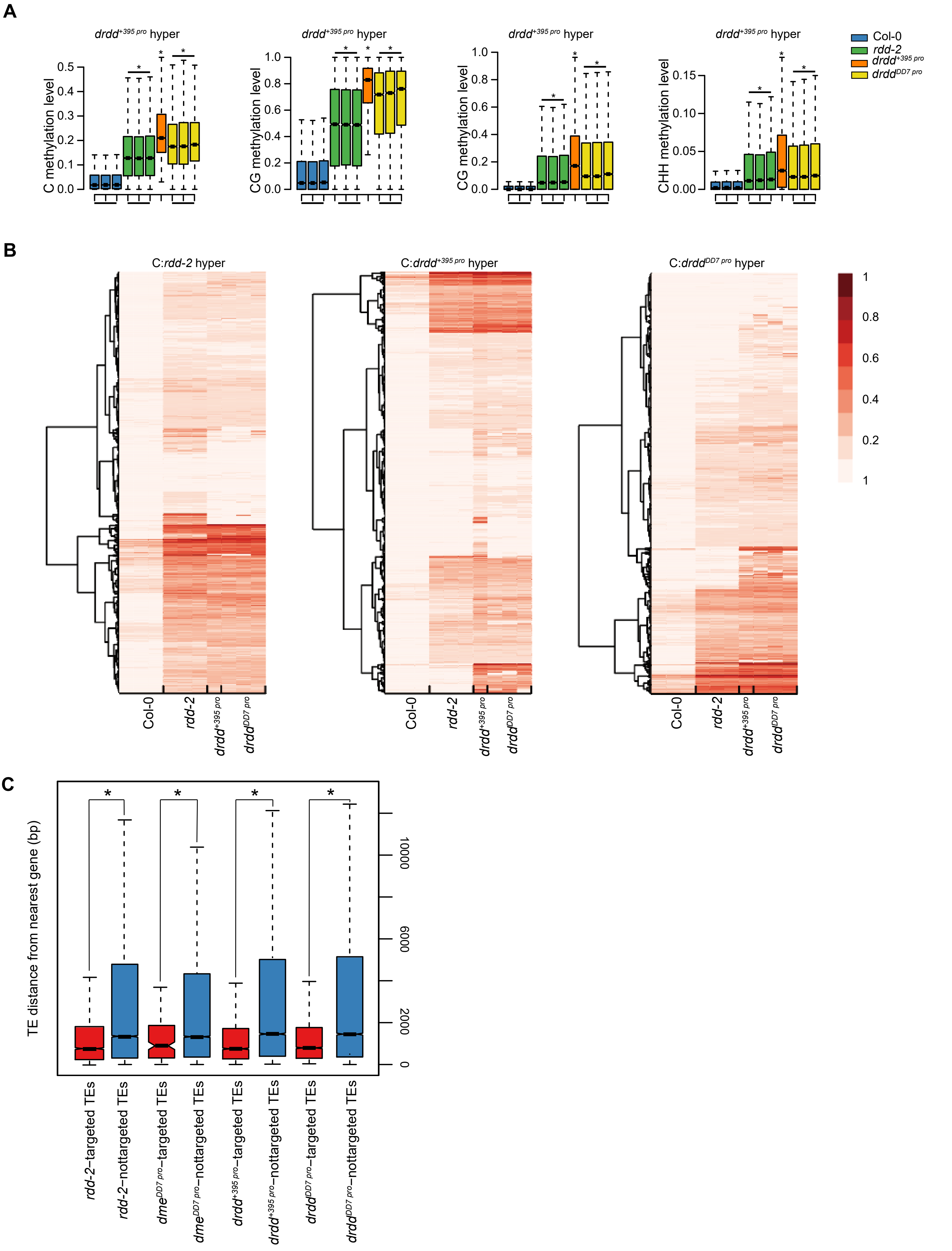

Supplementary Figure 4. Characterization of central cell-specific complementation *drdd* quarduple mutants.

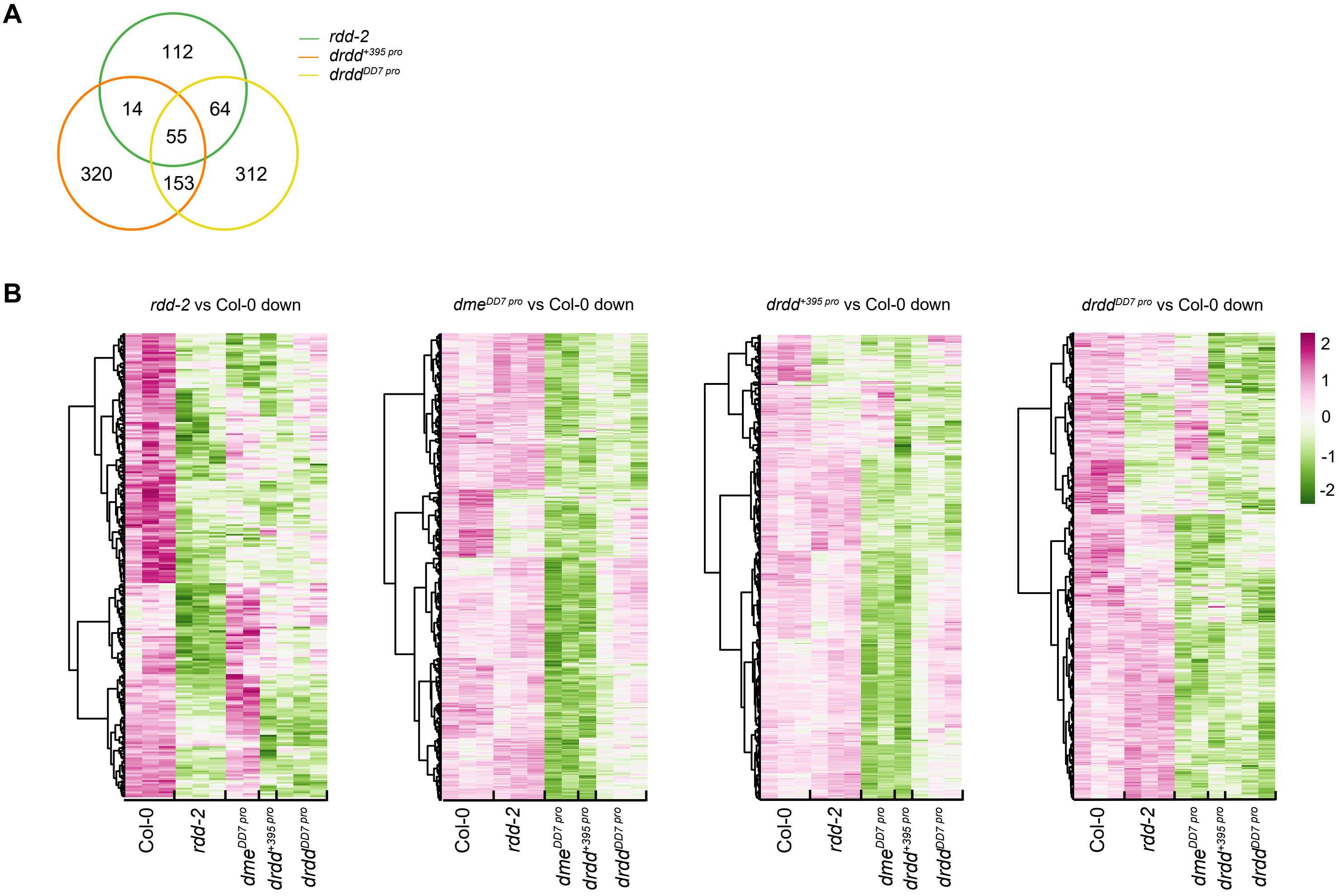

Supplementary Figure 5. Transcriptome analysis for *drdd* quadruple mutants.

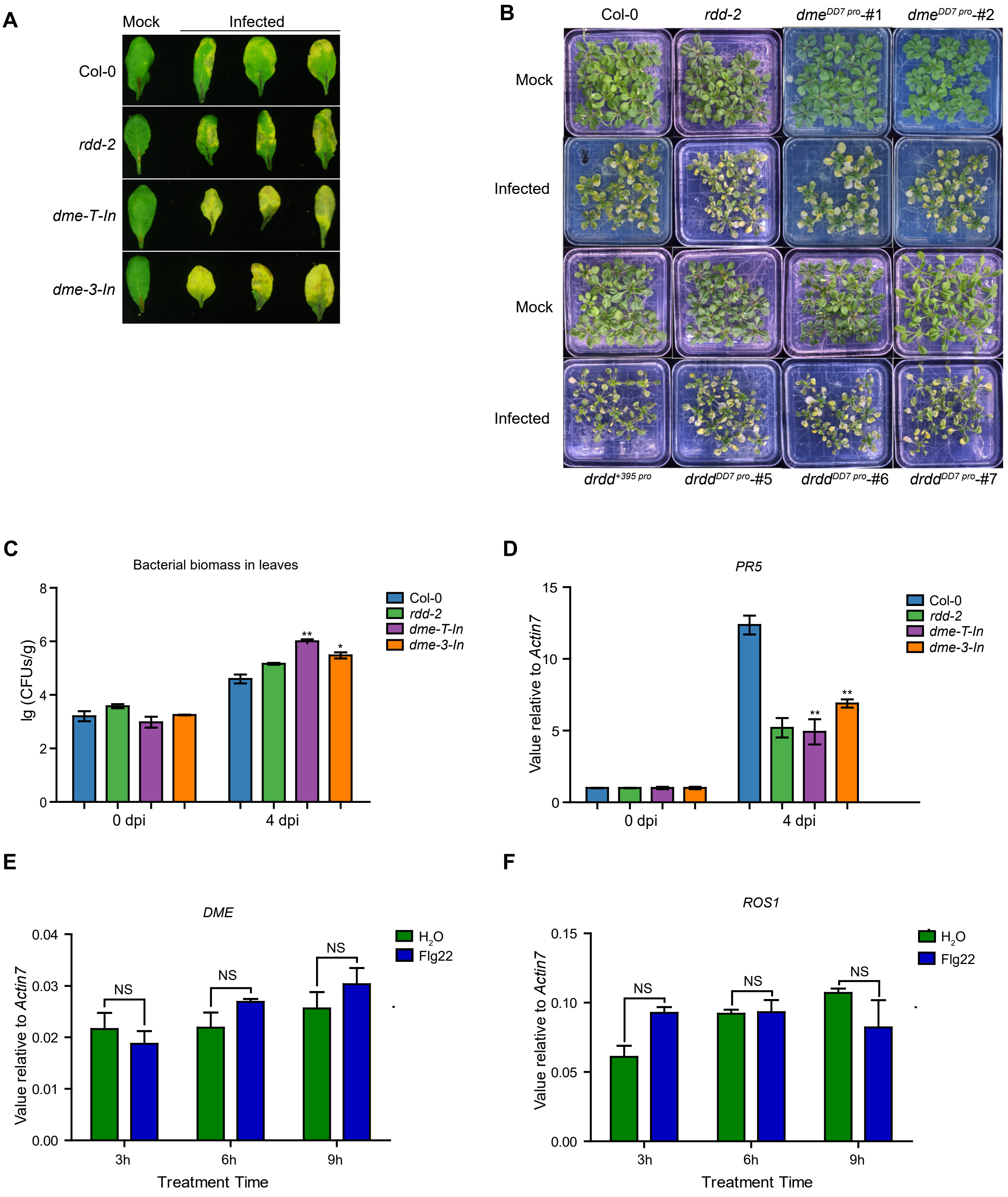

Supplementary Figure 6. ***Pst*** **DC3000 infection phenotype of** ***dme*** **weak-allele mutants.**

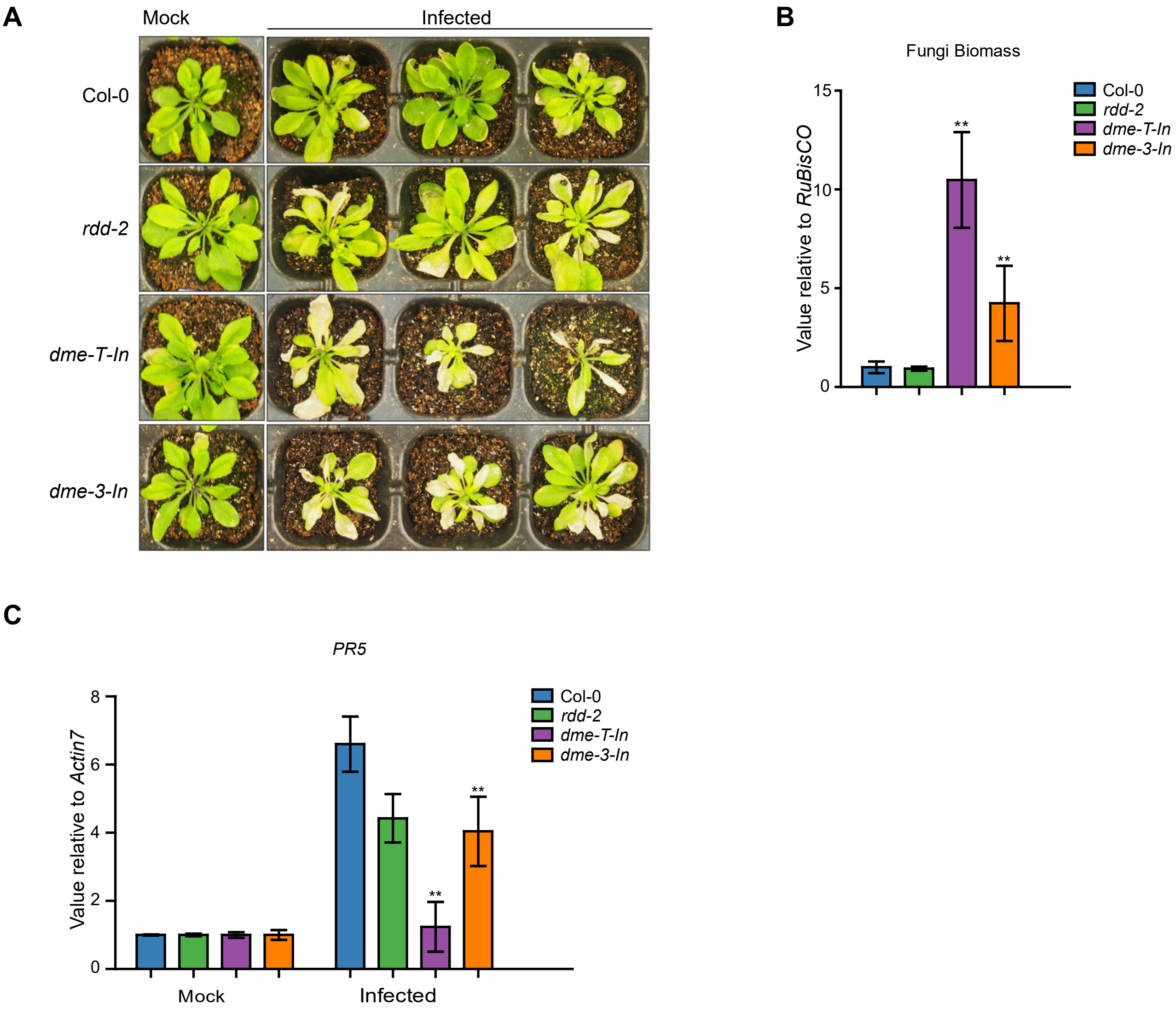

Supplementary Figure 7. *V. dahliae* infection phenotype of *dme* weak-allele mutants.

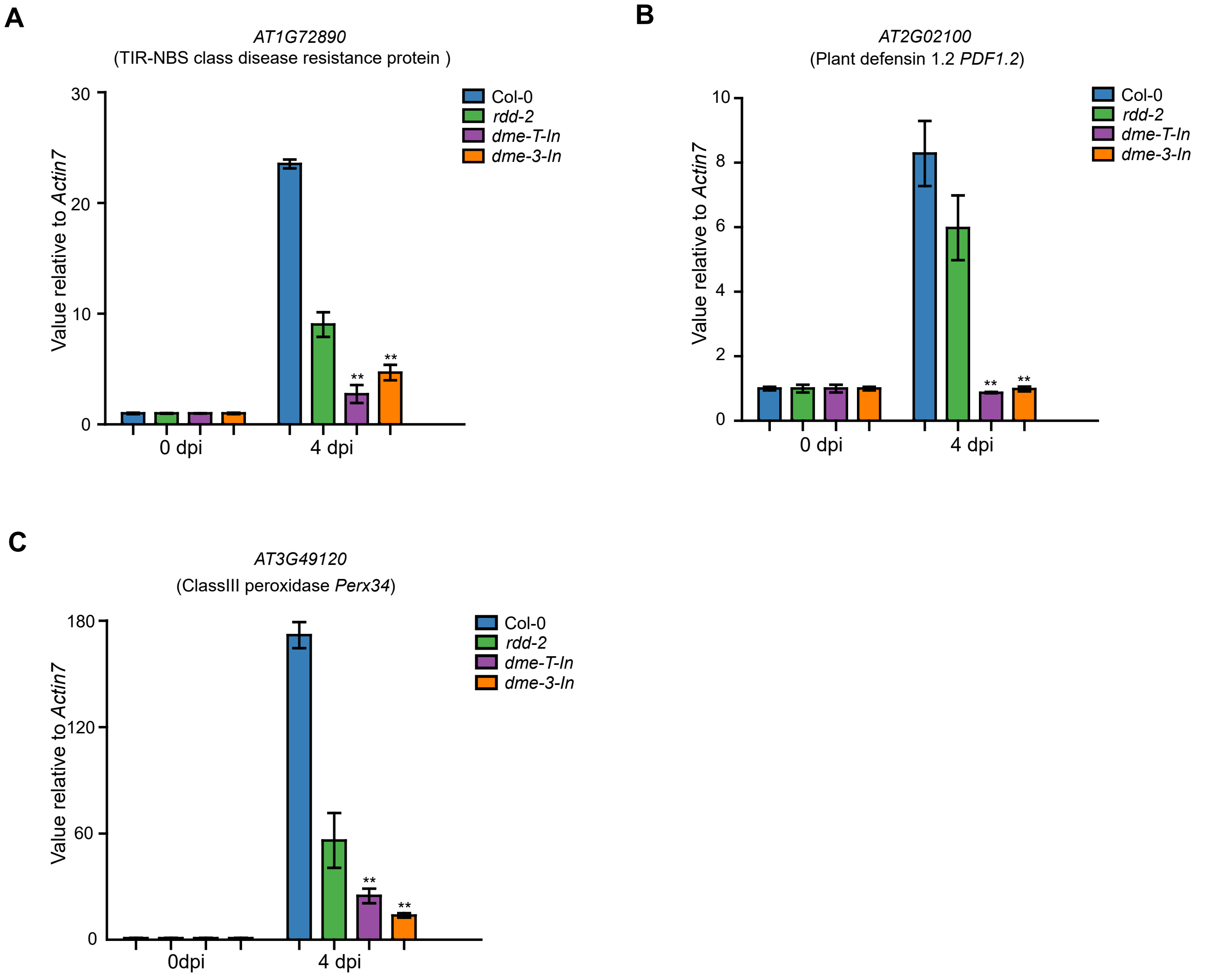

Supplementary Figure 8. Expression analysis for defense-related genes in *Pst* DC3000 infected *dme* weak-allele mutants.

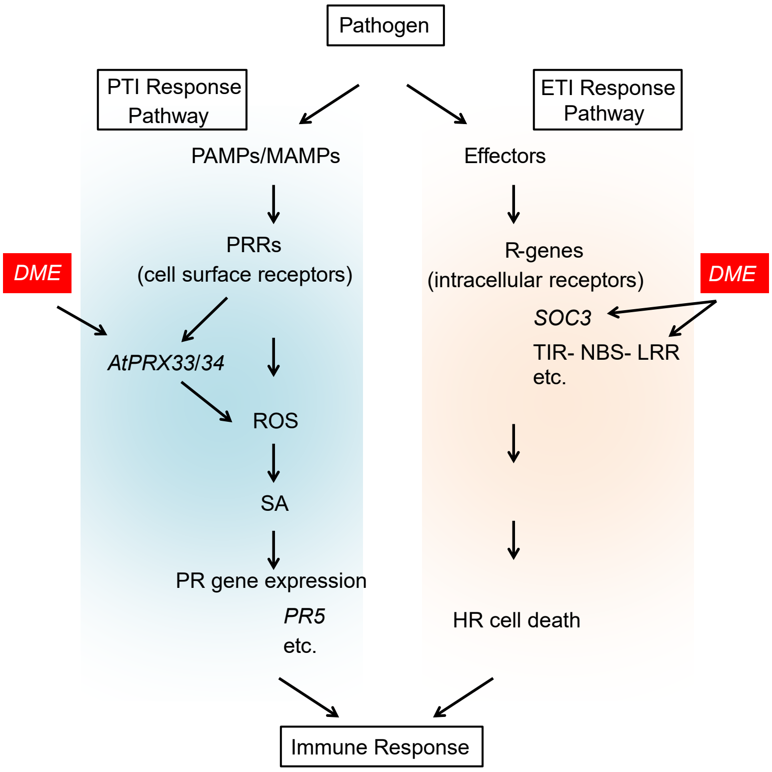

Supplementary Figure 9. A working model for DME function in disease resistance response.
